# Synthesis and In Vitro Evaluation as Potential Anticancer and Antioxidant Agents of Diphenylamine-Pyrrolidin-2-one-Hydrazone Derivatives

**DOI:** 10.3390/ijms242316804

**Published:** 2023-11-27

**Authors:** Irma Zubrickė, Ilona Jonuškienė, Kristina Kantminienė, Ingrida Tumosienė, Vilma Petrikaitė

**Affiliations:** 1 Department of Organic Chemistry, Kaunas University of Technology, Radvilėnų pl. 19, 50254 Kaunas, Lithuania; irmsanvaityte@gmail.com (I.Z.); ilona.jonuskiene@ktu.lt (I.J.); ingrida.tumosiene@ktu.lt (I.T.); 2 Department of Physical and Inorganic Chemistry, Kaunas University of Technology, Radvilėnų pl. 19, 50254 Kaunas, Lithuania; 3 Laboratory of Drug Targets Histopathology, Institute of Cardiology, Lithuanian University of Health Sciences, Sukilėlių pr. 13, 50162 Kaunas, Lithuania; 4 Institute of Biotechnology, Life Sciences Center, Vilnius University, Saulėtekio al. 7, 10257 Vilnius, Lithuania

**Keywords:** diphenylamine, pyrrolidin-2-one, melanoma, prostate tumor, triple-negative breast cancer, pancreatic tumor, 3D tumor model, migration, antioxidative, FRAP

## Abstract

The title compounds were synthesized by the reaction of 5-oxo-1-(4-(phenylamino)phenyl)pyrrolidine-3-carbohydrazide with various aldehydes bearing aromatic and heterocyclic moieties and acetophenones, and their cytotoxicity was tested via MTT assay against human triple-negative breast cancer MDA-MB-231, human melanoma IGR39, human pancreatic carcinoma Panc-1, and prostate cancer cell line PPC-1. Furthermore, the selectivity of compounds towards cancer cells compared to fibroblasts was also investigated. Four compounds were identified as the most promising anticancer agents out of a series of pyrrolidinone-hydrazone derivatives bearing a diphenylamine moiety. These compounds were most selective against the prostate cancer cell line PPC-1 and the melanoma cell lines IGR39, with EC_50_ values in the range of 2.5–20.2 µM against these cell lines. In general, the compounds were less active against triple-negative breast cancer MDA-MB-231 cell line, and none of them showed an inhibitory effect on the migration of these cells. In the ‘wound healing’ assay, *N*′-((5-nitrothiophen-2-yl)methylene)-5-oxo-1-(4-(phenylamino)phenyl)pyrrolidine-3-carbohydrazide was identified as the most promising derivative that could be further developed as an antimetastatic agent. *N*′-(5-chloro- and *N*′-(3,4-dichlorobenzylidene)-5-oxo-1-(4-(phenylamino)phenyl)pyrrolidine-3-carbohydrazides most efficiently reduced the cell viability in IGR39 cell spheroids, while there was no effect of the investigated pyrrolidinone-hydrazone derivatives on PPC-1 3D cell cultures. Antioxidant activity determined via FRAP assay of *N*′-(1-(4-aminophenyl)ethylidene)-5-oxo-1-(4-(phenylamino)phenyl)pyrrolidine-3-carbohydrazide was 1.2 times higher than that of protocatechuic acid.

## 1. Introduction

Cancer is a major life-threatening disease and a considerable barrier to increasing life expectancy [1]. In 2019, in 112 of 183 countries, cancer was the first or second leading cause of death among the general population within the age limit of 70 years. It is expected that by 2040 the global cancer burden will increase by 47% in comparison to 2020 [2]. In 2020, 18.1 million cancer cases had been estimated worldwide; 9.3 million of these cases were in men and 8.8 million were diagnosed for women. Breast cancer was one of the most often diagnosed cancers, making up 11.7% of all estimated cancer cases (2.3 million estimated new cases); the share of lung cancer was 11.4%, and colorectal cancer made up 10.0%, followed by prostate (7.3%) and stomach (5.6%) cancers [3].

The common cause of cancer is metastasis and failure of treatment approaches. The main treatment approaches are surgery, chemotherapy, radiotherapy, hormonal therapy, and immunotherapy. The distribution of anticancer drugs throughout most tissues and, thus, the combating of cancer cells in the process of metastases, is a benefit of chemotherapy [4].

Lipid peroxidation reactions as well as oxidation of DNA and proteins caused by reactive intermediates from oxidative stress are the main factors behind the carcinogenic process [5]. The overproduction of reactive species and the imbalance in the equilibrium between free radicals and antioxidants causes oxidative stress, which may be the reason for many pathologies. Increased concentrations of secondary messengers of oxidative stress, which are by-products of oxidative damage, have been detected in plasma and tissue related to lung, gastric, colon, and breast cancers [6]. There has been an increasing interest in using antioxidants for medical purposes in recent years. Antioxidant molecules have been shown to be one of the most versatile and effective forms of complementary anticancer therapy, as they integrate both therapeutic and preventive aspects [7].

**Figure 1:**
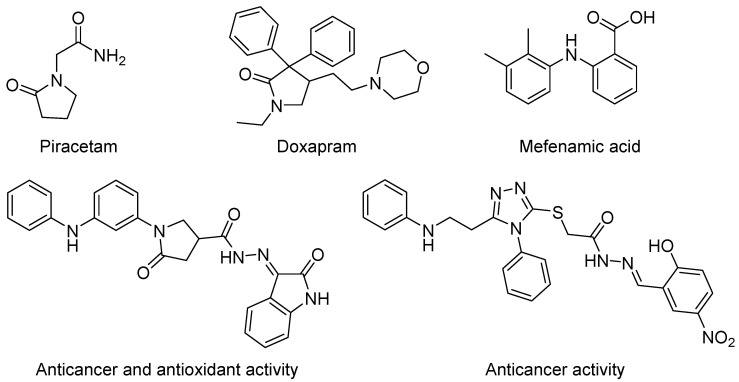
Commercial drugs and synthetic biologically active compounds [32,33] bearing pyrrolidin-one, diphenylamine, and hydrazone moieties.

The pyrrolidin-2-one scaffold is one of the essential nitrogen-containing pharmacophores present in approved commercial drugs (Figure 1). Piracetam (2-oxo-1-pyrrolidine acetamide) is a nootropic cyclic GABA derivative used in myoclonus, sickle cell disease, alcohol dependence, and as a general cognitive enhancer [8]. Doxapram (1-ethyl-4-(2-morpholin-4-ylethyl)-3,3-diphenyl-pyrrolidin-2-one) is used to treat patients with respiratory failure. Povidone–iodine, a chemical complex of polyvinylpyrrolidone and triiodide, is a topical antiseptic agent used for the treatment and prevention of wound infection [9].

The pyrrolidine moiety provides benefits in drug design because of the unconstrained conformation of the ring, which can be locked and tuned with the suitable substituents [10]. The pyrrolidin-2-one moiety incorporated into hybrid chemical structures has shown significant pharmacological properties, including those associated with anticancer activity [11]. 1-((2-Hydroxynaphthalen-1-yl)(phenyl)(methyl))pyrrolidin-2-one derivatives have been demonstrated to be potent PI3K inhibitors and anticancer agents [12]. 4-(4-Substituted benzylidene)-5-oxopyrrolidine-2-carboxylic acids and their derivatives bearing thiadiazole moiety have been shown to possess cytotoxic activity towards the MCF-7 cell line (19–108% cell viability at 1.0 nM), thus indicating their good anticancer activity [13]. A series of *tert*-butyl 3-cyano-3-cyclopropyl-2-oxopyrrolidine-4-carboxylates have been synthesized as macrocyclic Tyk2 inhibitors [14]. Jasiewicz et al. have demonstrated that the introduction of pyrrolidine moiety into the structures of thiocaffeine derivatives resulted in exceptionally active antioxidant compounds, for which efficiency of protection of human erythrocytes against AAPH-induced oxidative damage is of the same level as that of the standard antioxidant Trolox, indicating the significant cytoprotective potential [15].

Hydrazone derivatives are another significant class of biologically active compounds in pharmaceutical and medicinal chemistry. Their biological activity is conditioned by the presence of the active azomethine pharmacophore [16]. In hydrazones, the nitrogen atom is nucleophilic and the carbon has a both electrophilic and nucleophilic nature [17]. In addition, the physicochemical properties of the compounds are altered by the hydrazone moiety; moreover, it is able to release active components at lower pH. The development of a pH-sensitive drug delivery system using hydrazone linkers could benefit from the fact that the extracellular pH of the cancer cell is mostly acidic [18]. Thus, hydrazone derivatives, bearing various heterocyclic moieties, are of significant importance in medicinal chemistry owing to their diverse biological activities, such as anticancer, antioxidant, antibacterial, antifungal, anticonvulsant, anti-inflammatory, etc. [19,20,21,22,23,24,25].

Diphenylamine is a flexible skeleton for drug development [26]. Diphenylamine derivatives have been reported to possess important biological properties, which include antimicrobial, analgesic, anti-inflammatory, anticonvulsant [27], and anticancer activities. Shimizu et al. [28] have shown that the presence of a diphenylamine moiety enhanced the inhibitory activity in the autophosphorylation of FGF-R2 of a series of quinoline derivatives, among which 2-[[2-[[4-[4-[[4-(1,1-dimethylethyl)phenyl]amino]phenoxy]-6-methoxy-7-quinolinyl]oxy]ethyl]amino]ethanol has been identified as a potent inhibitor in autophosphorylation of FGF-R2 with IC_50_ value of 88 nM and inhibited the proliferation of the human scirrhous gastric carcinoma cell line, OCUM-2MD3, in a concentration-dependent manner. 2,4′-Bis diphenylamine hydrazones bearing thiadiazole, mercaptotriazole, and mercapto-oxadiazole moieties have been reported to possess the ability to inhibit EGFR tyrosine kinase. The reported compounds exhibited anticancer activity against breast cancer cell line MCF-7 with IC_50_ values in the range of 0.73–2.38 μM [29]. The antioxidant activity of diphenylamine derivatives depends on the secondary amine group. Diphenylamine has been shown to be an effective antioxidant in studies of lipid peroxidation [30].

In this context, based on the pharmacological properties of the moieties discussed above and as a continuation of our search for biologically active hybrid compounds with heterocyclic fragments [31,32,33], we report the synthesis of a series of 5-oxo-1-(4-(phenylamino)phenyl)pyrrolidine hydrazones bearing different aromatic and heterocyclic moieties and an investigation of their anticancer properties in 2D and 3D cell cultures, as well as their antioxidant activity.

## 2. Results and Discussion

### 2.1. Chemistry

**Scheme 1:**
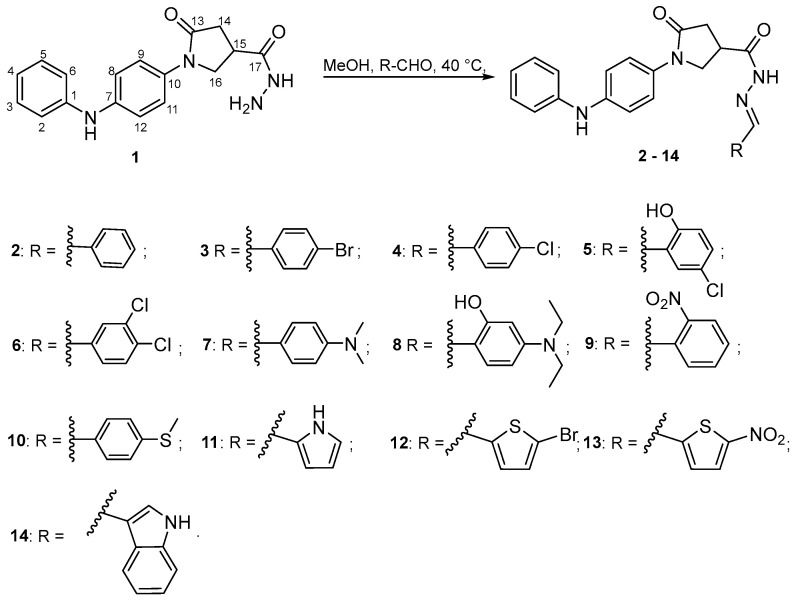
Synthesis of hydrazones **2**−**14**.

**Scheme 2:**
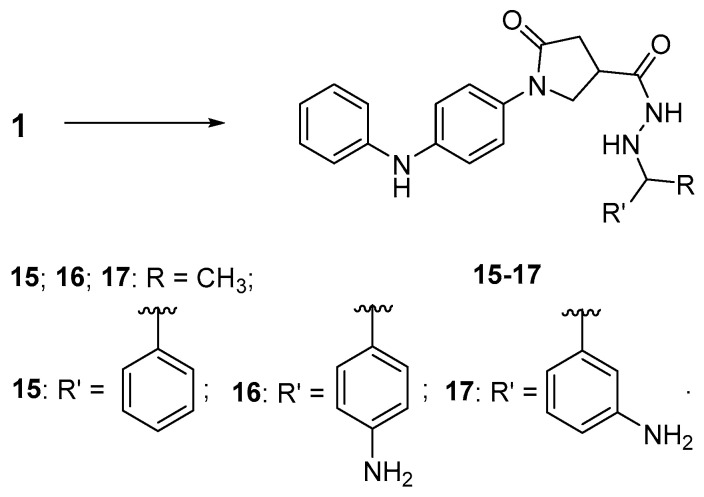
Synthesis of hydrazones **15**−**17**.

Hydrazones **2**–**17** were synthesized according to the synthesis route reported previously [31,32,33], as shown in Scheme 1 and Scheme 2. 

The starting compound, 5-oxo-1-(4-(phenylamino)phenyl)pyrrolidine-3-carbohydrazide (**1**), was synthesized as reported in [32]. Hydrazone derivatives **2**–**17** were synthesized from carbohydrazide **1** [32] and the corresponding aldehydes (**2**–**14**, Scheme 1) or acetophenones [34] (**15**–**17**, Scheme 2) in methanol at 60–70 °C in the yield range of 34–64%. The structures of the synthesized compounds have been confirmed using ^1^H and ^13^C NMR, IR, and HRMS data (Figures S1–S48). In the ^1^H NMR spectra of the target compounds **2**–**17**, the proton signals attributed to the protons of the amino group in the hydrazine moiety of **1** (4.31 ppm and 4.47 ppm) [32] have been replaced by the respective proton signals of the aromatic and heterocyclic moieties, which have originated from the respective aldehyde or ketone. In the ^1^H NMR spectra of all target hydrazones (Figures S1, S4, S7, S10, S13, S16, S19, S22, S25, S28, S31, S34, S37, S40, S43 and S46) protons of the benzene ring resonated in the aromatic region of the spectrum. In the ^1^H NMR of dimethylaminobenzene derivative **7** (Figure S16), the protons of two methyl groups resonated as two singlets at 2.95 ppm and 2.96 ppm, while the protons of the ethyl group in **8** resonated at 1.02–1.10 ppm (2CH_3_) and 3.35–3.43 ppm (2CH_2_) in the ^1^H NMR spectrum of **8** (Figure S22). In the ^1^H NMR of **10** (Figure S25), the protons of the methylthio group resonated as two singlets at 2.51 ppm and 2.55 ppm. In the ^1^H NMR spectrum for **11**, multiplets in the range of 5.85–7.48 ppm have been attributed to the protons of the pyrrole ring (Figure S28). In the ^1^H NMR spectra of 5-substituted thiophene derivatives **12** and **13**, the thiophene protons resonated as multiplets in the range of 7.24–7.48 ppm (Figures S31 and S34). In the ^1^H NMR spectra of **15–17** (Figures S40, S43 and S46), the resonance attributed to the methine group proton H_18_ is absent and the signals of the methyl group protons are present at 3.93 ppm (**15**), 2.13 ppm (**16**), and 2.18, 2.21 ppm (**17**), respectively. In the ^13^C NMR spectra of hydrazones **2**–**17** (Figures S2, S5, S8, S11, S14, S17, S20, S23, S26, S29, S32, S35, S38, S41, S44 and S47), the carbon resonances attributed to the carbonyl group carbons C_13_ and C_17_ are shifted downfield compared to the resonances of the respective carbon atoms in the ^13^C NMR spectrum of the starting compound **1** [32].

The ^1^H NMR spectra of hydrazones **3**–**14** display double sets of resonances of the NH-CO_2_ group proton and the adjacent methine proton H_18_ due to restricted rotation around the amide bond (Figures S4, S7, S10, S16, S19, S22, S25, S28, S31, S34 and S37). On the basis of the splitting of the proton resonances, it can be stated that in DMSO-*d_6_*, hydrazones exist as a mixture of *Z*/*E* isomers with respect to the hindered rotation around the amide bond. Generally, the *Z* isomer is a predominating one [31]. In the ^1^H NMR spectra, the resonances attributed to the NH protons of *Z* isomers are in a lower field with respect to the signals of the same protons in the *E* isomers [35].

### 2.2. Pharmacology

#### 2.2.1. Cytotoxicity

**Figure 2:**
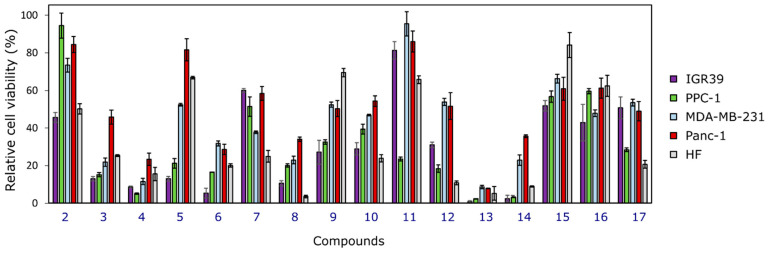
Effect of compounds **2**–**17** on cancer cell viability at 100 µM concentration against human melanoma IGR39, human prostate cancer PPC-1, human triple-negative breast cancer MDA-MB-231, human pancreatic carcinoma Panc-1 cell lines, and human fibroblasts HF, *n* = 3.

5-Oxo-1-(4-(phenylamino)phenyl)pyrrolidine-3-carbohydrazides exhibited different activity against tested cancer cell lines at 100 μM (Figure 2). This concentration was selected based on the solubility of the compound and also on our experience from the previous research, as 50–100 µM allows one to distinguish the most active compounds [33,36].

The majority of the tested hydrazone derivatives showed the lowest activity against human pancreatic cell lines Panc-1 and triple-negative breast cancer cell line MDA-MB-231, except compounds **2**, **7**, and **16** (Figure 2). This was not surprising, since these types of cancer are characterized by high resistance to many available chemotherapeutic drugs and are the most difficult to treat due to different molecular mechanisms [36,37]. The same observation was made in our previous research [33]. However, in this study, compound **13** bearing 5-nitrothiophene moiety has been identified as the potential candidate to be developed further as a drug against pancreatic and breast cancer, as it inhibited the cell viability of both types of cancer up to ~9%, compared to the control.

**Figure 3:**
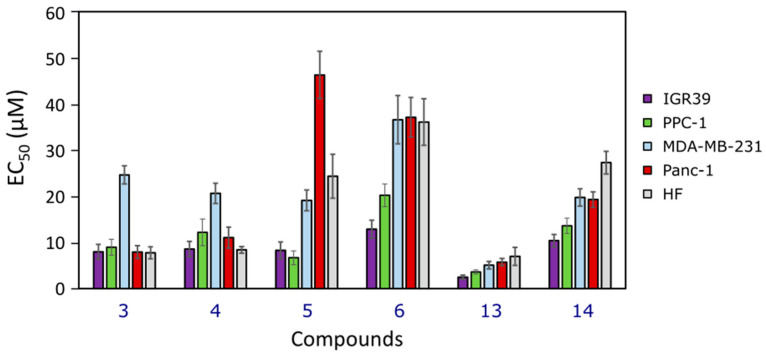
EC_50_ values of the most active compounds **3**, **4**, **5**, **6**, **13**, and **14** obtained by the MTT assay, *n* = 3. Incubation time with the compounds was 72 h.

The activity of hydrazone derivatives against melanoma IGR39 and prostate carcinoma PPC-1 cell lines was relatively higher, but variable among compounds. Some compounds showed the strongest effect on melanoma cells (compounds **2**, **3**, **5**, **6**, **8**, **9**, **10**, **13**, **14**, **15**, and **16**); meanwhile, others were most active against the prostate carcinoma cell line (compounds **4**, **11**, **12**, and **17**). It is interesting to note that **3,** bearing *p*-bromobenzylidene moiety, affected melanoma cells, while **4,** bearing *p*-chlorobenzylidene fragment, inhibited prostate carcinoma cells. Another pair that significantly affected cells of different cancer types is 5-bromothiophene derivative **12** (prostate carcinoma cells) and 5-nitrothiophene derivative **13** (melanoma cells). However, not all of the compounds identified as affecting cancer cells were more selective towards cancer cells compared to fibroblasts (normal cells). Compounds **2**, **7**, **8**, **10**, **12**, and **17** affected fibroblasts more than the tested cancer cell lines; therefore, they were excluded from further research as potentially cytotoxic substances. Compounds **9**, **11**, **15**, and **16** were not studied in more detail due to their relatively lower effect on cancer cell viability (they inhibited cell viability by less than 20%). In total, taking into account the above-mentioned rejection criteria and based on the highest activity and selectivity, six of the most promising hydrazone derivatives, namely **3**, **4**, **5**, **6**, **13**, and **14**, were selected for further studies, and their effective concentrations (EC_50_ values), which reduce cell viability by 50%, were determined (Figure 3). Among the synthesized compounds, **3**, **4**, **5**, and **6** are the ones bearing halogen-substituted benzene rings, whereas heterocyclic moieties, namely thiophene and indole fragments, make **13** and **14**, respectively, differ from other investigated compounds.

Unfortunately, after determination of the dose–effect correlation, compounds **3** and **4** bearing bromo- or chloro-substituted benzylidene moiety, respectively, were identified as nonselective against cancer cells (Figure 3). Due to this reason, they were excluded from other biological assays. Pyrrolidinone derivative **13** bearing 5-nitrothiophene moiety was the most active against all tested cell lines (EC_50_ = 2.50 ± 0.46 µM against IGR39, EC_50_ = 3.63 ± 0.45 µM against PPC-1, EC_50_ = 5.10 ± 0.80 µM against MDA-MB-231, and EC_50_ = 5.77 ± 0.80 µM against Panc-1 cell line). Its selectivity towards cancer cells compared to fibroblasts ranged from 2.8 (in IGR39) to 1.2 (in Panc-1). Another compound with higher selectivity towards all tested cancer cell lines (from 1.4 to 2.6) was indole derivative **14**, although its effect on cell viability was lower (EC_50_ ranged from 10.40 ± 1.35 µM in the melanoma cell line to 19.77 ± 1.86 µM in the triple-negative breast cancer cell line). Despite compounds **5** and **6** showing comparably lower activity out of the six selected hydrazone derivatives, they were identified as the most selective ones against prostate carcinoma (compound **5** was 3.6 times more selective towards this type of cancer compared to fibroblasts) and melanoma (compound **6** was 2.8 times more selective towards this type of cancer compared to fibroblasts). On the other hand, it is well known that cytotoxicity in the cell monolayer does not necessarily represent the real in vivo situation due to the lack of stroma cells that are present in tumors and could contribute to the development of resistance [38]. As we have already discussed in our previous research, it is also not well understood how the selectivity results should be interpreted [33,39]. Considering that selectivity towards cancer cells compared to fibroblasts could be evaluated as favorable when the cytotoxicity ratio is 2 or more, in our case, we identified at least four compounds (**5**, **6**, **13**, and **14**) that are relatively selective against the tested cancer cells. These compounds were chosen for further studies in migration assay and 3D cell culture model.

Also, it should be noted that all four compounds were more active than the anti-melanoma drug dacarbazine, which inhibits melanoma cell proliferation at only 25–100 μM [40]. Of course, there are also other much more active drugs that act at nanomolar concentrations with a well-established mechanism of action, which are already used to treat melanoma, such as BRAF inhibitor darafenib [41]. However, the druggability of compounds should be judged not only on the basis of the cytotoxicity and selectivity, but also on the basis of their potential to be active in multi-drug resistant diseases and other properties.

#### 2.2.2. Effect on Cell Migration

**Figure 4:**
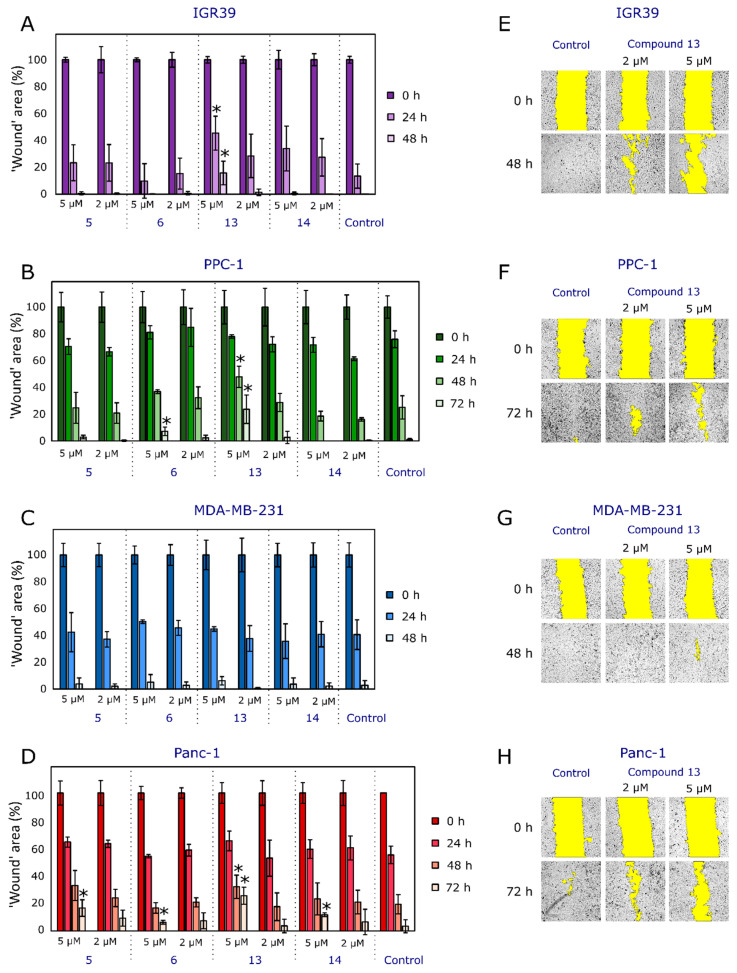
Effect of compounds **5**, **6**, **13**, and **14** on human malignant melanoma IGR39 (**A**), human prostate cancer PPC-1 (**B**), human triple-negative breast cancer MDA-MB-231 (**C**), and human pancreatic carcinoma (Panc-1) (**D**) cell migration, *n* = 3. Photos of ‘wound’ area (marked in yellow) in IGR39 (**E**), PPC-1 (**F**), MDA-MB-231 (**G**), and Panc-1 (**H**) monolayer at the beginning and the end of experiment. Scale bar indicates 100 µm. Asterisks (*) indicate *p* < 0.05 (untreated cells).

The effect of the four most active 5-oxo-1-(4-(phenylamino)phenyl)pyrrolidine-3-carbohydrazides on cell migration was assessed by ‘wound healing’ assay at the concentrations of 2 and 5 µM (Figure 4). The effect of 2 µM and 5 µM concentrations on cell viability was determined after 24 and 48 h on human melanoma IGR39 and triple-negative breast cancer MDA-MB-231 cell lines, and after 24, 48, and 72 h on human pancreatic carcinoma Panc-1 and prostate carcinoma PPC-1 cell lines (the description of the procedure (Procedure S1) and results (Figures S49–S52) are provided in Supplementary Material).

At both concentrations, all the compounds tested did not inhibit triple-negative breast cancer MDA-MB-231 cell migration compared to the control (*p* > 0.05) (Figure 4C). This type of cancer is characterized as a very invasive highly aggressive phenotype, and is generally very resistant to many available anticancer drugs and requires more personalized therapy [42]; thus, it was not surprising that the new synthesized compounds did not affect the migration of these cells. Additionally, none of the hydrazone derivatives affected the migration of melanoma, pancreatic, and prostate cancer cell lines at a concentration of 2 µM (Figure 4A,B,D). Compound **13** bearing 5-nitrothiophene moiety was the most active one, and it inhibited the migration of all cell lines except MDA-MB-231, especially after a longer incubation. The ‘wound’ area in Panc-1 cell monolayers in the presence of compound **13** was 7.5 times larger after 72 h of incubation compared to the control (Figure 4D,H) and ~24 times larger in PPC-1 monolayers after the same experiment time (Figure 4B,F). In the case of the IGR39 cell line, the control ‘wound’ was completely closed while its area was still (15.8 ± 8.8)% after 48 h of incubation with compound **13** (Figure 4A,E). The migration of the PPC-1 cell line was also inhibited by the higher concentration of compound **6** (Figure 4B), but only at the end of the experiment (72 h of incubation). The compounds mostly affected the ‘wound healing’ of the pancreatic cancer Panc-1 cell line—all of them reduced cell migration after 72 h of incubation at concentration of 5 µM (Figure 4D). This delayed and variable effect of compounds on migration could be explained by the possible inhibition of kinases, as in other studies cell motility was also lower after longer incubation [43], and the changes in signaling pathways could affect cell motility in several different ways [44].

In general, the correlation between cell viability and cell migration at 2 and 5 µM concentrations was not established; thus, it could be assumed that the ‘wound healing’ effects were not influenced directly by reduced cell proliferation. On the other hand, many not yet discovered compounds’ effects on cell division and metabolic pathways cannot be excluded. In summary, all four compounds did not reduce the cell viability by more than 20% after 48 h and 72 h of incubation (Figures S49–S52 in Supplementary Material).

#### 2.2.3. Compound Effect on Tumor Spheroid Growth and Their Viability

In cell monolayer, the tested compounds were most active and selective towards melanoma IGR39 and prostate carcinoma PPC-1 cell lines (Figure 3); thus, further experiments in 3D cultures focused on the effect on spheroid growth and viability that were made from these two cell lines.

Spheroids were made of cancer cells combined with fibroblast in a 1:1 ratio to better represent the real tumor microenvironment.

**Figure 5:**
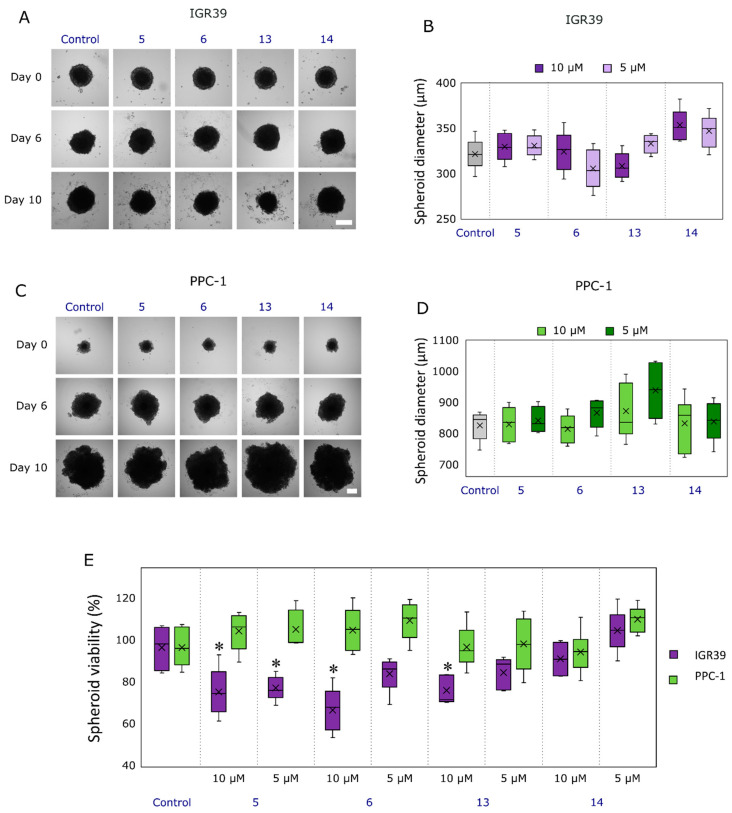
Effect of the most active compounds **5**, **6**, **13**, and **14** on 3D cell cultures. (**A**) Photos of human melanoma IGR39 tumor spheroids at the end of experiment (after 8 days of incubation with 10 µM of compounds). (**B**) IGR39 spheroid size at the end of experiment. (**C**) Photos of human prostate carcinoma PPC-1 tumor spheroids at the end of experiment (after 8 days of incubation with 10 µM of compounds). (**D**) PPC-1 spheroid size at the end of experiment. (**E**) Cell viability in IGR39 and PPC-1 spheroids. Asterisks (*) indicate *p* < 0.05 compared to the control (untreated spheroids), crosses (×) indicate means; inner dashes indicate medians; whiskers indicate maximum and minimum values. Scale bars indicates 200 µm.

At the beginning of the experiment, both types of spheroids were 200–220 µm in diameter (Figure 5A,C). However, IGR39 spheroids grew slower and retained a more oval shape, while PPC-1 spheroids grew faster and at the end of the experiment gained an irregular shape. Spheroid growth was affected differently by incubation with compounds, e.g., compound **5** did not affect spheroid growth, while compound **14** slightly reduced the growth of PPC-1 spheroids (Figure 5D), but IGR39 spheroids became somewhat larger (Figure 5B). Despite these variabilities among compounds, none of them at either concentration revealed a statistically significantly effect on the spheroid size (Figure 5B,D) (*p* > 0.05). Meanwhile, the effect on spheroid cell viability was different (Figure 5E). Compounds did not reduce the viability of PPC1 spheroids, even at a concentration of 10 µM, but three hydrazone derivatives (**5**, **6**, and **13**) showed a statistically significant effect on the viability of the IGR39 spheroid compared to the control. The most active was compound **5**, which reduced IGR39 spheroid cell viability up to ~75%.

Different observations among the results of spheroid size and spheroid size viability confirm the phenomenon already revealed by other scientists and our group, that those two parameters are not necessarily correlated [45,46], therefore, one should measure them in parallel to more accurately assess the compound activity in 3D cell cultures.

The differences in cell viability between selected cancer cell line spheroids could be partly explained by the established high expression of EGFR in IGR39 cell line [47]. However, prostate cancer cells are also characterized by an elevated EGFR level [48]. Although other researchers have shown that higher EGFR expression is related to the increased growth and invasion of prostate cancer cells in vitro [49], it is also known that the blockade of this receptor could induce compensation mechanisms, and alternative receptors could be expressed [50]. This phenomenon could reduce the efficacy of EGFR targeting molecules, and it could be the reason why the tested compounds were not active in both types of cell spheroids. Additionally, the results suggest that there could be some differences at the molecular level of mechanisms of action, too.

In summary, with regard to the chemical structure–activity relationship, one of the most active compounds, **5**, is the one containing chlorine and hydroxyl substituents in the benzene ring. It can be assumed that the combination of these two substituents had a positive effect on the selective activity of **5**, since mono chlorosubstituted benzylidene derivative **4** was identified as not being selective against the tested cancer cells. It can be noted that another compound bearing the disubstituted benzene ring with hydroxyl group substituent is **8**; however, both compounds **7** and **8**, containing a disubstituted amino group as a substituent, were excluded from further research as potentially cytotoxic substances. It is worth noting that the indole moiety present in the structure of **14** makes it different from the other synthesized compounds. Indole derivative **14** is the only one among the synthesized compounds bearing a bicyclic heterocyclic moiety, the presence of which has influenced the higher activity against melanoma and prostate cancer cells in vitro.

Considering the promising activity of the most active compounds **5**, **6**, **13**, and **14** in different assays, they are planned to be further developed as anticancer agents. Compound **13** should be developed as an antimetastatic agent by applying more advanced cell migration/invasion assays, such as single cell migration assays or invasion/migration assays, in a 3D microenvironment, considering the limitations of the “wound-healing” assay due to its well-known cell-neighboring effect, cell damage, etc. Compounds **5** and **6** should be further developed in more advanced melanoma models, investigating their more clinically translational effects in animals. However, before moving to more advanced models, detailed studies of the mechanism of action of those most active compounds would be needed by exploring their effects on specific cancer progression-related pathways in order to establish their targets in cancer cells. Simultaneously, more effective compounds will be further designed as potent anticancer agents based on the structure–activity relationship data gained for the most active compounds.

#### 2.2.4. Antioxidant Activity

The ferric reducing antioxidant power (FRAP) assay was used to evaluate the antioxidant potential of new compounds **2**–**17**. Antioxidant assays are based on determining reduction power reactions (e.g., reduction Fe^3+^ to Fe^2+^) or scavenging free radicals (DPPH, ABTS). The FRAP assay is based on the method of single electron transfer (SET), measuring the reduction in the complex of ferric ions (Fe^3+^)-ligand to the blue ferrous complex (Fe^2+^) [51]. This method uses 2,4,6-tripyridyl-*s*-triazine (TPTZ) as the linking ligand to the iron [52]. The antioxidant activity of the synthesized compounds was compared with that of the well-known antioxidant protocatechuic acid. Protocatechuic acid (3,4-dihydroxybenzoic acid, PCA) is a natural phytochemical antioxidant found in medicinal plants and exhibits antioxidant, anti-inflammatory, and antiviral activities due to its chemical structure [53]. PCA is a building block for pharmaceuticals and is widely used to produce many medicinal products such as veratridine (sodium channel inactivation inhibitor), erlotinib (antineoplastic), Picatin II (treatment of hepatitis B), itopride hydrochloride (gastrointestinal motility drug) [54]. PCA can also cause the induction of apoptosis of human leukemia cells [55]. Moreover, the antioxidant activity of PCA is based on the reduction in free radical formation via the up-regulation of genes encoding enzymes with neutralizing activities [56].

**Figure 6:**
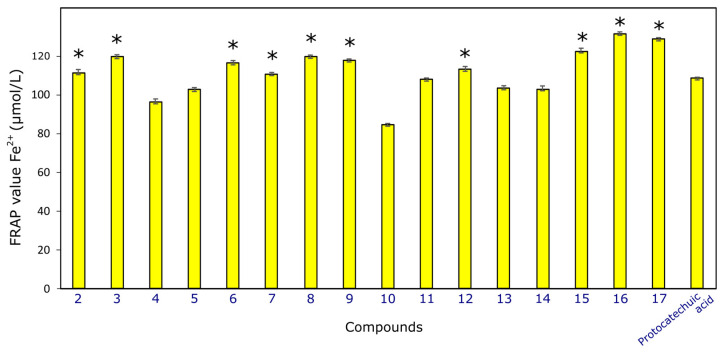
FRAP assay of **2**–**17** compounds and protocatechuic acid. Data are shown as mean ±SD from 3 experimental replicates. Asterisks (*) indicate *p* < 0.05 compared to the control (protocatechuic acid).

As seen from the FRAP assay data in Figure 6, almost all synthesized compounds demonstrated greater reduction of Fe^3+^ to Fe^2+^. The reducing power activity of compounds **16**, **17**, **15**, **3**, **8**, **9**, **6**, **2**, **12**, and **7** was higher than those of known antioxidants used as the controls. Compounds bearing *p*-aminophenylethylidene **16** (132.14 µmol/L), *m*-aminophenylethylidene **17** (129.28 µmol/L) and even unsubstituted phenylethylidene **15** (123.05 µmol/L) moieties have been identified as the most powerful reductants among the tested compounds. Hydrazone **17** derivative was among the most active compounds against the prostate carcinoma cell line, while **15** and **16** showed the strongest effect on melanoma cells. Hydrazones bearing *p*-bromobenzylidene **3** (120.08 µmol/L), *p*-(diethylamino)-o-hydroxybenzylidene **8** (120.24 µmol/L) and *o*-nitrobenzylidene **9** (118.32 µmol/L) fragments demonstrated a very high reducing power activity compared to that of protocatechuic acid (109.05 µmol/L). On the contrary to the results of the evaluation of anticancer activity, the antioxidant activity of nitrothiophene derivative **13** (103.95 µmol/L) was average compared to some other synthesized compounds. In general, no straightforward correlation has been detected between the anticancer and antioxidant activity of the tested compounds. It can be assumed that different mechanisms of action are most probably the reason [57].

## 3. Materials and Methods

### 3.1. Chemistry

#### 3.1.1. Chemical Reagents and Instruments

Reagents were bought from Sigma-Aldrich (St. Louis, MO, USA) and TCI Europe N.V. (Zwijndrecht, Belgium). The reaction course and purity of the synthesized compounds were monitored by TLC using aluminum plates precoated with silica gel 60 F254 (MerckKGaA, Darmstadt, Germany). The ^1^H and ^13^C NMR spectra were recorded in DMSO-*d_6_* on a Bruker Avance III (400 MHz, 101 MHz) spectrometer (Bruker BioSpin AG, Fällanden, Switzerland) operating in the Fourier transform mode. Chemical shifts (*δ*) are reported in parts per million (ppm) calibrated from TMS (0 ppm) as an internal standard for ^1^H NMR and DMSO-*d_6_* (39.43 ppm) for ^13^C NMR. FT-IR spectra (ν, cm^−1^) were recorded on a Perkin–Elmer Spectrum BX FT–IR spectrometer (Perkin–Elmer Inc., Waltham, MA, USA) using KBr pellets. Mass spectra were obtained on a Bruker maXis UHR-TOF mass spectrometer (Bruker Daltonics, Bremen, Germany) with positive ESI ionization. The melting points were determined on a MEL-TEMP (Electrothermal, A Bibby Scientific Company, Burlington, NJ, USA) melting point apparatus and are uncorrected.

#### 3.1.2. 5-Oxo-1-(4-(phenylamino)phenyl)pyrrolidine-3-carbohydrazide (**1**) Was Synthesized as Described in [32]

M.p., ^1^H and ^13^C NMR spectra were found to be identical with the ones described in [32].

#### 3.1.3. General Procedure for the Synthesis of Compounds **2**–**17**

To hydrazide **1** (1.5 mmol) dissolved in methanol (25 mL), a corresponding aldehyde or ketone (2.5 mmol) was added followed by addition of concentrated HCl (2–3 drops). The reaction mixture was stirred at 60–70 °C for 20 min–4 h. The precipitate formed was filtered off, dried and recrystallized from methanol.

*N′-benzylidene-5-oxo-1-(4-(phenylamino)phenyl)pyrrolidine-3-carbohydrazide* (**2**), Prepared from benzaldehyde. Yield 47% (0.36 g), light blue crystals; m.p. 122–123 °C. IR (KBr) ν_max_ (cm^−1^): 1604, 1683 (C=O), 3023, 3311 (NH); ^1^H NMR (400 MHz, DMSO-*d_6_*): 2.63–2.81 (m, 2H, H_14_), 3.89–3.93 (m, 1H, H_15_), 3.99–4.04 (m, 2H, H_16_), 6.79 (t, 1H, *J* = 7.2 Hz, H_4_), 7.01–7.07 (m, 4H, H_2,6,8,12_), 7.20 (t, 2H, *J* = 7.2 Hz, H_3,5_), 7.43–7.45 (m, 2H, H_Ar″_), 7.49–7.51 (m, 5H, H_Ar″_), 7.86–7.87 (m, 1H, H_18_), 8.12 (s, 1H, NH), 8.68 (s, 1H, NH); ^13^C NMR (101 MHz, DMSO-*d_6_*): δ 35.37 (C_15_), 35.51 (C_14_), 50.75, 52.81 (C_16_), 116.91, 117.31, 117.65, 120.20, 121.95, 128.96, 129.12, 129.55, 129.81, 130.26, 131.96, 132.06, 134.21, 140.67, 144.04, 162.16 (C_Ar,Ar′,Ar″_+C_18_), 171.74, 173.97 (C_13,17_). HRMS (ESI+): m/z calcd for C_24_H_22_N_4_O_2_ 399.1822 [M+H]^+^, found 399.1708.
*N′-(4-bromobenzylidene)-5-oxo-1-(4-(phenylamino)phenyl)pyrrolidine-3-carbohydrazide* (**3**), Prepared from 4-bromobenzaldehyde. Yield 44% (0.37 g), green crystals; m.p. 198–199 °C. IR (KBr) ν_max_ (cm^−1^): 1653, 1687 (C=O), 3113, 3397 (NH); ^1^H NMR (400 MHz, DMSO-*d_6_*): 2.65–2.84 (m, 2H, H_14_), 3.89–3.93 (m, 1H, H_15_), 3.99–4.10 (m, 2H, H_16_), 6.79 (t, 1H, *J* = 7.2 Hz, H_4_), 7.00–7.07 (m, 4H, H_2,6,8,12_), 7.20 (t, 2H, *J* = 7.2 Hz, H_3,5_), 7.46 (d, 2H, *J* = 7.2 Hz, H_9,11_), 7.59–7.65 (m, 4H, H_Ar″_), 7.99 (s, 0.6H, H_18_), 8.11 (s, 1H, NH), 8.15 (s, 0.4H, H_18_), 11.55 (s, 0.6H, NH), 11.76 (s, 0.4H, NH); ^13^C NMR (101 MHz, DMSO-*d_6_*): δ 33.20, 35.03 (C_15_), 35.22, 35.75 (C_14_), 50.82, 51.18 (C_16_), 116.65, 116.69, 117.49, 121.67, 121.72, 123.53, 123.85, 129.14, 129.38, 129.59, 131.85, 131.93, 132.20, 133.62, 133.67, 140.35, 140.39, 143.10, 143.86, 146.46, 169.50 (C_Ar,Ar′,Ar″_+C_18_), 171.77, 172.02, 174.26 (C_13,17_). HRMS (ESI+): m/z calcd for C_24_H_21_BrN_4_O_2_ 477.0927 [M+H]^+^, found 477.0926.
*N′-(4-chlorobenzylidene)-5-oxo-1-(4-(phenylamino)phenyl)pyrrolidine-3-carbohydrazide* (**4**), Prepared from 4-chlorobenzaldehyde. Yield 59% (0.4 g), black crystals; m.p. 209–210 °C. IR (KBr) ν_max_ (cm^−1^): 1655, 1689 (C=O), 3113, 3398 (NH); ^1^H NMR (400 MHz, DMSO-*d_6_*): 1.85–1.92 (m, 2H, H_14_), 3.04–3.11 (m, 1H, H_15_), 3.21–3.25 (m, 2H, H_16_), 5.91 (t, 1H, *J* = 7.2 Hz, H_4_), 6.06–6.19 (m, 4H, H_2,6,8,12_), 6.33 (t, 2H, *J* = 7.6 Hz, H_3,5_), 6.60–6.65 (m, 4H, H_9,11,Ar″_), 6.86 (d, 2H, *J* = 8.0 Hz, H_Ar″_), 7.15 (s, 0.7H, H_18_), 7.26 (s, 1H, NH), 7.34 (s, 0.3H, H_18_), 10.76 (s, 0.7H, NH), 10.83 (s, 0.3H, NH); ^13^C NMR (101 MHz, DMSO-*d_6_*): δ 32.89, 34.74 (C_15_), 34.92, 35.50 (C_14_), 50.31, 50.73 (C_16_), 116.20, 116.24, 117.18, 117.21, 119.38, 121.05, 121.10, 128.55, 128.73, 128.92, 129.18, 131.75, 131.83, 133.10, 134.33, 134.57, 139.82, 139.86, 142.37, 143.68, 145.72, 168.90, 171.16 (C_Ar,Ar′,Ar″_+C_18_), 171.36, 173.74 (C_13,17_); HRMS (ESI+): m/z calcd for C_24_H_21_ClN_4_O_2_ 433.1432 [M+H]^+^, found 433.1428.
*N′-(5-chloro-2-hydroxybenzylidene)-5-oxo-1-(4-(phenylamino)phenyl)pyrrolidine-3-carbohydrazide* (**5**), Prepared from 5-chlorosalicylaldehyde. Yield 51% (0.37 g), light yellow crystals; m.p. 223–224 °C. IR (KBr) ν_max_ (cm^−1^): 1628, 1674 (C=O), 2855, 3401 (NH); ^1^H NMR (400 MHz, DMSO-*d_6_*): 2.67–2.76 (m, 2H, H_14_), 3.67–3.68 (m, 1H, H_15_), 3.94–4.07 (m, 2H, H_16_), 6.92 (t, 1H, *J* = 7.2 Hz, H_4_), 6.98 (d, 2H, *J* = 7.2 Hz, H_2,6_), 7.00 (d, 2H *J* = 7.2 Hz, H_8,12_), 7.38 (t, 2H *J* = 7.2 Hz, H_3,5_), 7.42 (d, 2H *J* = 7.2 Hz, H_9,11_), 7.69 (d, 2H, *J* = 7.6 Hz, H_Ar″_), 7.74 (m, 2H, H_18,Ar″_), 8.92 (s, 1H, NH), 11.10 (s, 1H, NH), 12.05 (s, 1H, OH); ^13^C NMR (101 MHz, DMSO-*d_6_*): δ 33.31 (C_15_), 35.14 (C_14_), 51.08 (C_16_), 116.25, 117.19, 118.48, 118.56, 119.95, 120.14, 121.08, 121.69, 121.96, 123.15, 123.24, 128.10, 128.68, 129.85, 132,02, 132.71, 133.10, 153.37, 157.30; 157.37, 160.92, 161,25, 162.94, 163.60 (C_Ar,Ar′,Ar″_+C_18_), 171.71, 174.37 (C_13,17_); HRMS (ESI+): m/z calcd for C_24_H_21_ClN_4_O_3_ 449.1381 [M+H]^+^, found 449.1377.
*N′-(3,4-dichlorobenzylidene)-5-oxo-1-(4-(phenylamino)phenyl)pyrrolidine-3-carbohydrazide* (**6**), Prepared from 3,4-dichlorobenzaldehyde. Yield 41% (0.29 g), light blue crystals; m.p. 220–221 °C. IR (KBr) ν_max_ (cm^−1^): 1603, 1675 (C=O), 2937, 3343 (NH); ^1^H NMR (400 MHz, DMSO-*d_6_*): 2.68–2.77 (m, 2H, H_14_), 3.86–3.90 (m, 1H, H_15_), 3.94–4.12 (m, 2H, H_16_), 6.24 (s, 2H, H_Ar″_), 6.79 (t, 1H, *J* = 7.2 Hz, H_4_), 7.01–7.07 (m, 4H, H_2,6,8,12_), 7.20 (t, 2H, *J* = 7.2 Hz, H_3,5_), 7.43–7.48 (m, 3H, H_9,11,Ar″_), 8.11 (s, 1H, NH), 8.18 (s, 0.7H, H_18_), 8.33 (s, 0.3H, H_18_), 11.14 (s, 0.7H, NH), 11.32 (s, 0.3H, NH). ^13^C NMR (101 MHz, DMSO-*d_6_*): δ 34.17, 34.87 (C_15_), 35.24, 35.88 (C_14_), 50.95, 51.35 (C_16_), 91.35, 91.41, 104.08, 116.69, 117.48, 119.96, 121.65, 121.77, 129.60, 131.92, 132.01, 139.52, 140.37, 143.86, 160.22, 162.53 (C_Ar,Ar′,Ar″_+C_18_), 172.33, 173.46 (C_13,17_); HRMS (ESI+): m/z calcd for C_24_H_20_Cl_2_N_4_O_2_ 489.0860 [M+Na]^+^, found 489.2140.
*N′-(4-(dimethylamino)benzylidene)-5-oxo-1-(4-(phenylamino)phenyl)pyrrolidine-3-carbohydrazide* (**7**), Prepared from 4-(dimethylamino)benzaldehyde. Yield 49% (0.34 g), light red crystals; m.p. 218–219 °C. IR (KBr) ν_max_ (cm^−1^): 1597, 1674 (C=O), 3077, 3335 (NH); ^1^H NMR (400 MHz, DMSO-*d_6_*): 2.68–2.79 (m, 2H, H_14_), 2.95, 2.96 (2s, 6H, 2CH_3_), 3.89–4.12 (m, 3H, H_15,16_), 6.73 (t, 1H, *J* = 7.2 Hz, H_4_), 6.74–6.81 (m, 2H, H_Ar″_), 7.03–7.09 (m, 4H, H_2,6,8,12_), 7.21 (t, 2H, *J* = 7.2 Hz, H_3,5_), 7.47–7.56 (m, 4H, H_9,11, Ar″_), 7.91 (s, 0.6H, H_18_), 8.07 (s, 0.4H, H_18_), 8.14 (s, 1H, NH), 11.28 (s, 0.6H, NH), 11.34 (s, 0.4H, NH); ^13^C NMR (101 MHz, DMSO-*d_6_*): δ 33.74 (C_15_), 35.60 (C_14_), 39.65, 39.77 (CH_3_), 50.47, 50.89 (C_16_), 111.08, 111.84, 116.19, 117.22, 119.37, 121.02, 121.08, 121.54, 124.53, 128.14, 128.43, 129.18, 131.55, 131.89, 139.78, 143.68, 144.42, 147.82, 151.37, 154.21 (C_Ar,Ar′,Ar″_+C_18_), 171.50, 173.02 (C_13,17_); HRMS (ESI+): m/z calcd for C_26_H_27_N_5_O_2_ 442.2244 [M+H]^+^, found 442.2241.
*N′-(4-(diethylamino)-2-hydroxybenzylidene)-5-oxo-1-(4-(phenylamino)phenyl)pyrrolidine-3-carbohydrazide* (**8**), Prepared from 4-(diethylamino)salicylaldehyde. Yield 42% (0.31 g), light yellow crystals; m.p. 239–240 °C. IR (KBr) ν_max_ (cm^−1^): 1629, 1676 (C=O), 3004, 3256 (NH), 3423 (OH); ^1^H NMR (400 MHz, DMSO-*d_6_*): 1.02–1.10 (m, 6H, CH_3_), 2.71–2.81 (m, 2H, H_14_), 3.35–3.43 (m, 4H, CH_2_), 4.02–4.08 (m, 3H, H_15,16_), 6.52 (s, 1H, H_Ar″_), 6.61 (d, 1H, *J* = 8.8 Hz, H_Ar″_), 6.79 (t, 1H, *J* = 7.2 Hz, H_4_), 7.01–7.08 (m, 4H, H_2,6,8,12_), 7.20 (t, 2H, *J* = 7.2 Hz, H_3,5_), 7.39–7.47 (m, 3H, H_9,11,Ar″_), 8.24 (s, 0.3H, H_18_), 8.31 (s, 0.7H, H_18_), 11.29 (s, 0.3H, NH), 11.44 (s, 0.7H, NH), 11.92 (s, 1H, OH); ^13^C NMR (101 MHz, DMSO-*d_6_*): δ 11.93 (CH_3_), 35.00 (C_15_), 35.74 (C_14_), 44.57 (CH_2_), 51.22 (C_16_), 40,56 (CH_3_), 104.96, 107.91, 116.70, 116.74, 117.48, 119.98, 121.70, 121.75, 129.61, 131.85, 131.94, 140.41, 143.86, 149.10, 159.62, 163.75, 169.03 (C_Ar,Ar′,Ar″_+C_18_), 171.81, 172.06 (C_13,17_). HRMS (ESI+): m/z calcd for C_28_H_31_N_5_O_3_ 486.2506 [M+H]^+^, found 486.2510.
*N′-(2-nitrobenzylidene)-5-oxo-1-(4-(phenylamino)phenyl)pyrrolidine-3-carbohydrazide* (**9**), Prepared from 2-nitrobenzaldehyde. Yield 39% (0.32 g), light red crystals; m.p. 192–193 °C. IR (KBr) ν_max_ (cm^−1^): 1596, 1676 (C=O), 3056, 3342 (NH); ^1^H NMR (400 MHz, DMSO-*d_6_*): 1.87–1.96 (m, 2H, H_14_), 3.07–3.11 (m, 1H, H_15_), 3.15–3.25 (m, 2H, H_16_), 5.92 (t, 1H, *J* = 7.4 Hz, H_4_), 6.16–6.22 (m, 4H, H_2,6,8,12_), 6.34 (t, 2H, *J* = 7.4 Hz, H_3,5_), 6.65 (d, 2H, *J* = 8.8 Hz, H_Ar″_), 6.76–6.93 (m, 2H, H_Ar″_), 7.16–7.25 (m, 2H, H_9,11_), 7.27 (s, 1H, NH), 7.63–7.80 (m, 2H, H_Ar″_), 7.55 (s, 0.7H, H_18_), 7.77 (s, 0.3H, H_18_), 10.98 (s, 0.7H, NH), 11.12 (s, 0.3H, NH); ^13^C NMR (101 MHz, DMSO-*d_6_*): δ 32.89 (C_15_), 34.72 (C_14_), 50.23 (C_16_), 116.21, 117.19, 119.39, 121.10, 124.54, 128.12, 128.36, 128.68, 129.18, 129.44, 130.51, 131.80, 132.16, 133.55, 133.93, 139.15, 139.84, 142.55, 143.66, 148.06, 148.88, 158.70, 169.15 (C_Ar,Ar′,Ar″_+C_18_), 171.29, 173.96 (C_13,17_); HRMS (ESI+): m/z calcd for C_24_H_21_N_5_O_4_ 444.1673 [M+H]^+^, found 444.1672.
*N′-(4-(methylthio)benzylidene)-5-oxo-1-(4-(phenylamino)phenyl)pyrrolidine-3-carbohydrazide* (**10**), Prepared from 4-(methylthio)benzaldehyde. Yield 58% (0.41 g), light red crystals; m.p. 193–194 °C. IR (KBr) ν_max_ (cm^−1^): 1597, 1660 (C=O), 3241, 3311 (NH); ^1^H NMR (400 MHz, DMSO-*d_6_*): 2.51, 2.55 (2s, 3H, CH_3_), 2.67–2.81 (m, 2H, H_14_), 3.92–3.98 (m, 1H, H_15_), 4.00–4.13 (m, 2H, H_16_), 6.80 (t, 1H, *J* = 7.0 Hz, H_4_), 7.03–7.10 (m, 4H, H_2,6,8,12_), 7.22 (t, 2H, *J* = 7.0 Hz, H_3,5_), 7.31 (d, 2H, *J* = 7.0 Hz, H_9,11_), 7.52 (d, 2H, *J* = 8.0 Hz, H_Ar″_), 7.64 (d, 2H, *J* = 8.0 Hz, H_Ar″_), 8.00 (s, 0.7H, H_18_), 8.15 (s, 1H, NH), 8.18 (s, 0.3H, H_18_), 11.54 (s, 0.7H, NH), 11.61 (s, 0.3H, NH); ^13^C NMR (101 MHz, DMSO-*d_6_*): δ 14.30 (CH_3_), 32.93 (C_15_), 34.74 (C_14_), 50.37 (C_16_), 116.20, 117.21, 119.38, 121.04, 121.09, 125.68, 127.31, 127.51, 129.19, 129.86, 130.60, 131.85, 139.81, 140.73, 141.05, 143.26, 143.68, 146.63, 168.68 (C_Ar,Ar′,Ar″_+C_18_), 171.40, 173.53 (C_13,17_); HRMS (ESI+): m/z calcd for C_25_H_24_N_4_O_2_S 445.1699 [M+H]^+^, found 445.1695.
*N′-((1H-pyrrol-2-yl)methylene)-5-oxo-1-(4-(phenylamino)phenyl)pyrrolidine-3-carbohydrazide* (**11**), Prepared from pyrrole-2-carboxaldehyde. Yield 34% (0.29 g), black crystals; m.p. 234–235 °C. IR (KBr) ν_max_ (cm^−1^): 1613, 1671 (C=O), 2926, 3108, 3400 (NH); ^1^H NMR (400 MHz, DMSO-*d_6_*): 2.63–2.78 (m, 2H, H_14_), 4.00–4.13 (m, 1H, H_15_), 4.15–4.17 (m, 2H, H_16_), 5.85–5.88 (m, 1H, H_Pyrrole_), 6.03–6.12 (m, 1H, H_Pyrrole_), 6.21–6.28 (m, 1H, H_Ar′_), 6.41–6.47 (m, 1H, H_Ar′_), 6.79 (t, 1H, *J* = 7.0 Hz, H_4_), 6.92–7.48 (m, 7H, H_Ar′,Pyrrole_), 7.84 (s, 0.6H, H_18_), 8.01 (s, 1H, NH), 8.11 (s, 0.4H, H_18_), 11.19 (s, 0.4H, NH), 11.33 (s, 0.6H, NH), 11.44 (s, 1H, NH); ^13^C NMR (101 MHz, DMSO-*d_6_*): δ 32.93 (C_15_), 34.74 (C_14_), 50.37 (C_16_), 116.20, 117.21, 119.38, 121.04, 121.09, 125.17, 125.68, 127.31, 127.51, 129.19, 129.86, 130.60, 131.85, 139.81, 140.73, 143.26, 143.68, 146.63, 168.68 (C_Ar,Ar′,Pyrrole_+C_18_), 171.40, 173.53 (C_13,17_); HRMS (ESI+): m/z calcd for C_22_H_21_N_5_O_2_ 388.1774 [M+H]^+^, found 388.1767.
*N′-((5-bromothiophen-2-yl)methylene)-5-oxo-1-(4-(phenylamino)phenyl)pyrrolidine-3-carbohydrazide* (**12**), Prepared from 5-bromo-2-thiophenecarboxaldehyde. Yield 56% (0.36 g), light green crystals; m.p. 240–241 °C. IR (KBr) ν_max_ (cm^−1^): 1596, 1670 (C=O), 2926, 3384 (NH); ^1^H NMR (400 MHz, DMSO-*d_6_*): 2.63–2.81 (m, 2H, H_14_), 3.87–4.06 (m, 3H, H_15,16_), 6.78 (t, 1H, *J* = 7.2 Hz, H_4_), 7.01–7.06 (m, 4H, H_2,6,8,12_), 7.19 (t, 2H, *J* = 7.2 Hz, H_3,5_), 7.24–7.29 (m, 1H, H_Thiophene_); 7.42–7.46 (m, 3H, H_9,11,Thiophene_), 7.83 (s, 0.5H, NH), 8.09 (s, 0.4H, H_18_), 8.22 (s, 0.3H, H_18_), 8.31 (s, 0.3H, H_18_), 8.70 (s, 0.5H, NH), 9.77 (s, 0.4H, NH), 11.54 (s, 0.3H, NH), 11.70 (s, 0.3H, NH); ^13^C NMR (101 MHz, DMSO-*d_6_*): δ 33.34 (C_15_), 35.16, 35.30 (C_14_), 52.59 (C_16_), 114.65, 115.27, 116.66, 116.69, 117.42, 117.46, 119.95, 121.69, 121.72, 124.00, 129.57, 130.46, 131.19, 132.94, 139.02, 143.84, 145.12, 154.77, 155.84 (C_Ar,Ar′,Ar″_+C_18_), 171.50, 173.73 (C_13,17_); HRMS (ESI+): m/z calcd for C_22_H_19_BrN_4_O_2_S 483.0491 [M+H]^+^, found 483.0483.
*N′-((5-nitrothiophen-2-yl)methylene)-5-oxo-1-(4-(phenylamino)phenyl)pyrrolidine-3-carbohydrazide* (**13**), Prepared from 5-nitro-2-thiophenecarboxaldehyde. Yield 43% (0.31 g), light blue crystals; m.p. 235–236 °C. IR (KBr) ν_max_ (cm^−1^): 1648, 1693 (C=O), 3080, 3414 (NH); ^1^H NMR (400 MHz, DMSO-*d_6_*): 2.65–2.82 (m, 2H, H_14_), 3.89–3.96 (m, 1H, H_15_), 3.99–4.06 (m, 2H, H_16_), 6.78 (t, 1H, *J* = 7.2 Hz, H_4_), 6.93–7.07 (m, 4H, H_2,6,8,12_), 7.19 (t, 2H, *J* = 7.2 Hz, H_3,5_), 7.42–7.48 (m, 4H, H_9,11,Thiophene_), 8.04 (t, 1H, *J* = 7.2 Hz, NH), 8.15 (s, 0.6H, H_18_), 8.40 (s, 0.4H, H_18_), 11.87 (s, 0.6H, NH), 12.03 (s, 0.4H, NH); ^13^C NMR (101 MHz, DMSO-*d_6_*): δ 33.34 (C_15_), 34.99, 35.33 (C_14_), 50.70, 51.05 (C_16_), 116.68, 117.45, 119.96, 119.98, 121.71, 129.43, 129.58, 130.05, 130.91, 131.88, 137.37, 140.40, 141.02, 143.84, 146.82, 150.91, 151.29, 169.82 (C_Ar,Ar′,Thiophene_+C_18_), 171.84, 174.33 (C_13,17_); HRMS (ESI+): m/z calcd for C_22_H_19_N_5_O_4_S 450.1234 [M+H]^+^, found 450.1232.
*N′-((1H-indol-3-yl)methylene)-5-oxo-1-(4-(phenylamino)phenyl)pyrrolidine-3-carbohydrazide* (**14**), Prepared from indole-3-carboxaldehyde. Yield 52% (0.36 g), red crystals; m.p. 238–239 °C. IR (KBr) ν_max_ (cm^−1^): 1612, 1660 (C=O), 3060, 3310, 3614 (NH); ^1^H NMR (400 MHz, DMSO-*d_6_*): 2.73–2.92 (m, 2H, H_14_), 3.96–4.04 (m, 1H, H_15_), 4.10–4.19 (m, 2H, H_16_), 6.76–6.81 (m, 1H, H_4_), 7.00–7.49 (m, 12H, H_2,3,5,6,8,9,11,12,Indole_), 7.74–7.77 (m, 1H, H _Indole_), 8.12, 8.14 (2s, 0.6H, NH), 8.21, 8.24 (2s, 1H, H_18_), 8.38 (s, 0.4H, NH), 11.16 (s, 0.6H, NH), 11.36 (s, 0.4H, NH), 11.55 (s, 1H, NH); ^13^C NMR (101 MHz, DMSO-*d_6_*): δ 33.27 (C_15_), 35.16 (C_14_), 51.00 (C_16_), 111.71, 112.25, 112.39, 116.67, 117.51, 119.96, 120.91, 121.20, 121.71, 122.21, 123.13, 124.35, 124.60, 129.60, 130.78, 130.91, 132.00, 137.34, 137.44, 140.36, 141.97, 143.87, 144.86, 168.50 (C_Ar,Ar′,Indole_+C_18_), 172.01, 173.34 (C_13,17_); HRMS (ESI+): m/z calcd for C_26_H_23_N_5_O_2_ 438.1931, found 438.1931 [M+H]^+^.
*5-Oxo-1-(4-(phenylamino)phenyl)-N′-(1-phenylethylidene)pyrrolidine-3-carbohydrazide* (**15**), Prepared from acetophenone. Yield 47% (0.31 g), light blue crystals; m.p. 225–226 °C. IR (KBr) ν_max_ (cm^−1^): 1678, 1730 (C=O), 3349, 3376 (NH); ^1^H NMR (400 MHz, DMSO-*d_6_*): 2.90–3.08 (m, 2H, H_14_), 3.93 (s, 3H, CH_3_), 4.16–4.30 (m, 3H, H_15,16_), 7.06 (t, 1H, *J* = 7.2 Hz, H_4_), 7.28–7.35 (m, 4H, H_2,6,8,12_), 7.47 (t, 2H, *J* = 7.2 Hz, H_3,5_), 7.57–7.93 (m, 6H, H_9,11,Acetophenone_), 8.14–8.19 (m, 2H, NH+H_Acetophenone_), 8.39 (s, 1H, NH); ^13^C NMR (101 MHz, DMSO-*d_6_*): δ 15.08 (CH_3_), 35.17 (C_15_), 35.31 (C_14_), 50.54 (C_16_), 116.70, 117.44, 119.99, 121.74, 126.82, 128.85, 129.60, 130.19, 131.75, 138.16, 140.46, 143.83, 157.65 (C_Ar,Ar′,Acetophenone_+C_18_), 171.53, 173.77 (C_13,17_); HRMS (ESI+): m/z calcd for C_25_H_24_N_4_O_2_ 413.1968 [M+H]^+^, found 413.1765.
*N′-(1-(4-aminophenyl)ethylidene)-5-oxo-1-(4-(phenylamino)phenyl)pyrrolidine-3-carbohydrazide* (**16**), Prepared from 4-aminoacetophenone. Yield 56% (0.36 g), dark blue crystals; m.p. 230–231 °C. IR (KBr) ν_max_ (cm^−1^): 1680, 1730 (C=O), 3349, 3376, 3400 (NH); ^1^H NMR (400 MHz, DMSO-*d_6_*): 2.13 (s, 3H, CH_3_), 2.65–2.81 (m, 2H, H_14_), 3.89–3.93 (m, 1H, H_15_), 3.95–4.04 (m, 2H, H_16_), 5.42 (s, 2H, NH_2_), 6.55–6.57 (m, 2H, H_Acetophenone_), 6.79 (t, 1H, *J* = 7.2 Hz, H_4_), 7.01–7.07 (m, 4H, H_2,6,8,12_), 7.20 (t, 2H, *J* = 7.2 Hz, H_3,5_), 7.44 (d, 2H, *J* = 8.8 Hz, H_9,11_), 7.64–7.66 (m, 2H, H_Acetophenone_), 8.11 (s, 1H, NH), 10.18 (s, 0.4H, NH), 10.40 (s, 0.6H, NH); ^13^C NMR (101 MHz, DMSO-*d_6_*): δ 26.18 (CH_3_), 34.39 (C_15_), 35.17, 35.31 (C_14_), 50.55 (C_16_), 112.94, 116.71, 117.44, 117.50, 120.00, 121.66, 121.75, 125.20, 127.66, 129.61, 131.02, 131.75, 140.40, 140.47, 143.83, 153.97 (C_Ar,Ar′, Acetophenone_+C_18_), 171.55, 173.78 (C_13,17_); HRMS (ESI+): m/z calcd for C_25_H_25_N_5_O_2_ 428.2087 [M+H]^+^, found 428.2083.
*N′-(1-(3-aminophenyl)ethylidene)-5-oxo-1-(4-(phenylamino)phenyl)pyrrolidine-3-carbohydrazide* (**17**), Prepared from 3-aminoacetophenone. Yield 64% (0.42 g), black crystals; m.p. 239–240 °C. IR (KBr) ν_max_ (cm^−1^): 1596, 1665 (C=O), 3094, 3184, 3397 (NH); ^1^H NMR (400 MHz, DMSO-*d_6_*): 2.18, 2.21 (2s, 3H, CH_3_), 2.66–2.86 (m, 2H, H_14_), 3.91–3.96 (m, 1H, H_15_), 4.00–4.12 (m, 2H, H_16_), 6.60–6.64 (m, 1H, H_Acetophenone_), 6.79 (t, 1H, *J* = 7.2 Hz, H_4_), 6.94 (d, 1H, *J* = 8.00 Hz, H_Acetophenone_), 7.01–7.49 (m, 12H, H_2,6,8,12,3,5,9,11,Acetophenone_+NH_2_), 8.11 (s, 1H, NH), 10.57 (s, 1H, NH); ^13^C NMR (101 MHz, DMSO-*d_6_*): δ 14.04, 14.76 (CH_3_), 33.52 (C_15_), 35.04, 35.16 (C_14_), 50.98 (C_16_), 111.89, 112.22, 113.21, 114.70, 115.10, 115.56, 115.80, 116.68, 117.51, 119.97, 121.68, 121.74, 129.33, 129.61, 131.93, 139.03, 140.38, 143.87, 148.73, 154.09, 169.90 (C_Ar,Ar′,Acetophenone_+C_18_), 172.18, 174.90 (C_13,17_); HRMS (ESI+): m/z calcd for C_25_H_25_N_5_O_2_ 428.2087 [M+H]^+^, found 428.2083.

### 3.2. Pharmacology

#### 3.2.1. Cell Culturing

The human malignant melanoma cell line IGR39, human triple-negative breast cancer MDA-MB-231, and human pancreatic carcinoma cell line Panc-1 were obtained from the American Type Culture Collection (ATCC, Manassas, VA, USA). Human foreskin fibroblasts (HF) CRL-4001 were originally obtained from ATCC and kindly provided by Prof. Helder Santos (University of Helsinki, Finland). Primary prostate carcinoma PPC-1 cell line was kindly provided by Prof. Tambet Teesalu (University of Tartu, Estonia). All cell lines were cultured in Dulbecco’s Modified Eagle’s GlutaMAX medium (Gibco (Carlsbad, CA, USA)), supplemented with 10,000 U/mL penicillin, 10 mg/mL streptomycin (Gibco), and 10% fetal bovine serum (Gibco). Cell cultures were grown at 37 °C in a humidified atmosphere containing 5% CO_2_. They were used until the passage of 20.

#### 3.2.2. Cell Viability Assay

The effect of synthesized compounds on cell viability was studied using 3-(4,5-dimethylthiazol-2-yl)-2,5-diphenyltetrazolium bromide (MTT; Sigma-Aldrich Co., St Louis, MO, USA) assay, as described elsewhere [58]. To summarize, the cells were seeded in triplicates in 96-well Corning plates (IGR39, MDA-MB-231, Panc-1 and PPC1: 4 × 10^3^ cells/well; HF: 5 × 10^3^ cells/well). The cells were treated with 100 μM of tested compounds after 24 h of incubation. After 72 h, the MTT reagent was added and the formazan crystals that had formed were dissolved in DMSO (Sigma-Aldrich Co., St. Louis, MO, USA). The absorbance was determined with a multidetection microplate reader at 570 and 630 nm.

Using the same MTT procedure, the EC_50_ values of the most active hydrazones, namely **3**, **4**, **5**, **6**, **13**, and **14,** were determined. The compound serial dilutions ranging from 50 µM to 1.56 µM were introduced to the cells. The Hill equation was used to calculate the EC_50_ value, or the concentration of a compound that results in a 50% reduction in the metabolic activity of cells.

#### 3.2.3. ‘Wound Healing’ Assay

‘Wound healing’ assay was employed to assess the ability of the most active hydrazones **5**, **6**, **13**, and **14** to inhibit cell migration, as published elsewhere [47]. Cancer cells were seeded at a density of 6 × 10^4^ cells/well in 24-well plates. After 48 h of incubation, a 100 µL pipette tip was used to make the scratch in each well. Following a single PBS wash, the fresh medium containing 2 and 5 µM of compounds **5**, **6**, **13**, and **14** was added to the cells. As a negative control, medium containing 0.1% DMSO was employed. 

#### 3.2.4. Compound Activity in Cell 3D Cultures (Spheroids)

The magnetic 3D Bioprinting method was utilized to form cancer cell spheroids as described elsewhere [59]. In short, the cells at 70% confluency were incubated with Nanoshuttle (n3D Biosciences, Inc., Houston, TX, USA) for 8 h. After that, the cells were trypsinized, centrifuged and seeded into ultra-low attachment 96-well plate at a ratio of 1.5 × 10^3^ cancer cells and 1.5 × 10^3^ human fibroblasts/well. The plate was incubated for two days at 37 °C in a humidified atmosphere on a magnetic drive. Next, the medium with 5 and 10 µM of selected compounds was added. Photos of spheroids were taken every two days using an Olympus IX73 inverted microscope (OLYMPUS CORPORATION, Tokyo, Japan), and analysis of spheroid size was performed using ImageJ, version 1.53o (National Institutes of Health, Bethesda, MD, USA) and Microsoft Office Excel 2016 software (Microsoft Corporation, Redmond, WA, USA).

On the last day of experiment, 10 µL of WST-1 reagent (Sigma-Aldrich Co, St. Louis, MO, USA) was added to each well. Following 10-h incubation, 50 µL of liquid from each well was moved to another 96-well plate and the absorbance was measured at 460 and 530 nm.

#### 3.2.5. Antioxidant Activity Determined by FRAP Assay 

Antioxidant activity was determined as an increase in absorbance at 593 nm, and the results were expressed as of Fe^2+^ μmol/L and in relation to antioxidants, and protocatechuic acid, as positive control [60]. The FRAP reagent contained 2.5 mL of 10 mM TPTZ solution in 40 mM HCl as well as 2.5 mL of FeCl_3_ (20 mM) and 25 mL of acetate buffer (0.3 M, pH = 3.6). Then, 100 μL of analyzed compounds (20 mM) were mixed with 3 mL of the FRAP reagent. The absorbance of the reaction mixture was measured spectrophotometrically at 593 nm using a UV-1280 UV-VIS spectrophotometer (Shimadzu Corporation, Kyoto, Japan). To compose the calibration curve, five concentrations of FeSO_4_·7H_2_O (5, 10, 15, 20, 25 μM) were used, and the absorbance was measured as a sample solution.

#### 3.2.6. Statistical Analysis

All biological experiments were repeated at least three times, calculating the mean and standard deviation. The data were processed using Microsoft Office Excel 2016 software (Microsoft Corporation, Redmond, WA, USA). Statistical analysis was performed by using Student’s *t*-test. The level of significance was set as *p* < 0.05.

## 4. Conclusions

In conclusion, a series of novel hydrazone derivatives were synthesized in the reactions of 5-oxo-1-(4-(phenylamino)phenyl)pyrrolidine-3-carbohydrazide with various aldehydes bearing aromatic and heterocyclic moieties or acetophenones, and their anticancer properties were evaluated.

Compounds **5**, **6**, **13**, and **14** were identified as the most promising anticancer agents out of a series of pyrrolidinone-hydrazone derivatives. They were most selective against melanoma cell lines IGR39 and prostate cancer cell line PPC-1, and their EC50 values against these cell lines were in the range of 2.5–20.2 µM. Most of the tested compounds showed lower activity against triple-negative breast cancer MDA-MB-231 cell line, and none of them showed an inhibiting effect on the migration of those cells. In the ‘wound healing’ assay, the most promising compound was compound **13**, and it could be further developed as antimetastatic agent. The compounds **5** and **6** most efficiently reduced the cell viability in IGR39 cell spheroids, while there was no effect of the tested hydrazone derivatives in PPC-1 3D cell cultures. Compound **16** was the most powerful reducing agent in the FRAP assay.

## Data Availability

Data are contained within this article.

## Supplementary Materials

The following supporting information can be downloaded at: https://www.mdpi.com/1422-0067/24/23/16804/s1.
