# Supplementary material for: Synthesis and In Vitro Evaluation as Potential Anticancer and Antioxidant Agents of Diphenylamine-Pyrrolidin-2-one-Hydrazone Derivatives
Source: Int J Mol Sci. 2023 Nov 27;24(23):16804. doi: 10.3390/ijms242316804 (PMC10871122; doi:10.3390/ijms242316804)
Supplement: Supplementary file 1 [file ijms-24-16804-s001.zip › ijms-2729031-supplementary.pdf]

Supplementary Material

Synthesis and In Vitro Evaluation as Potential Anticancer and Antioxidant Agents of Diphenylamine-Pyrrolidin-2-one-Hydrazone Derivatives

Irma Zubrickė [1], Ilona Jonuškienė [1], Kristina Kantminienė [2*], Ingrida Tumosienė [1], and Vilma Petrikaitė [3,4*]

[1] Department of Organic Chemistry, Kaunas University of Technology, Radvilėnų pl. 19, 50254 Kaunas, Lithuania;
[2] Department of Physical and Inorganic Chemistry, Kaunas University of Technology, Radvilėnų pl. 19, 50254 Kaunas, Lithuania;
[3] Laboratory of Drug Targets Histopathology, Institute of Cardiology, Lithuanian University of Health Sciences, Sukilėlių pr. 13, 50162 Kaunas, Lithuania;
[4] Institute of Biotechnology, Life Sciences Center, Vilnius University, Saulėtekio al. 7, 10257 Vilnius, Lithuania

* Correspondence: vilma.petrikaite@gmail.com (VP); kristina.kantminiene@ktu.lt (KK)

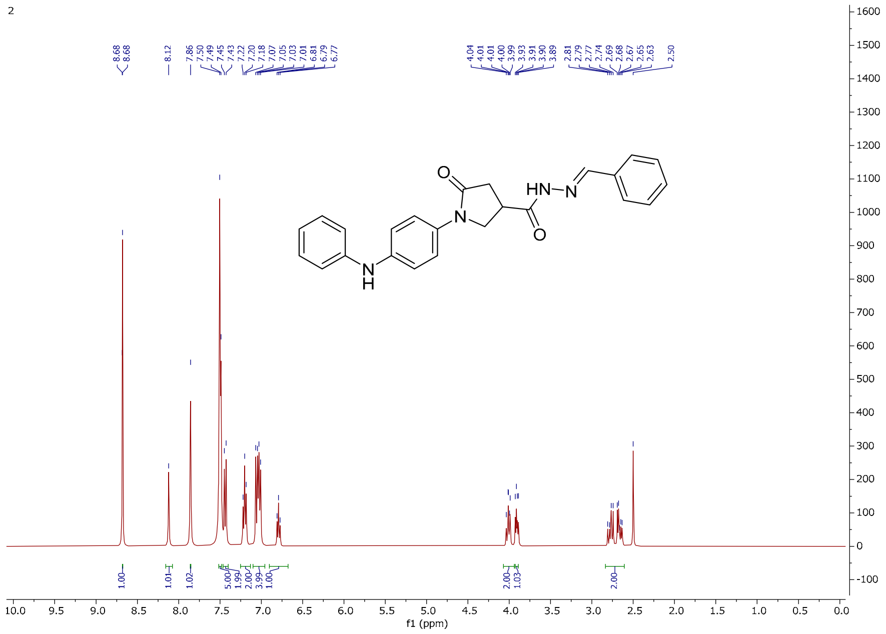

Figure S1. ^{1}H NMR (400 MHz, DMSO-d6) spectrum of **2**

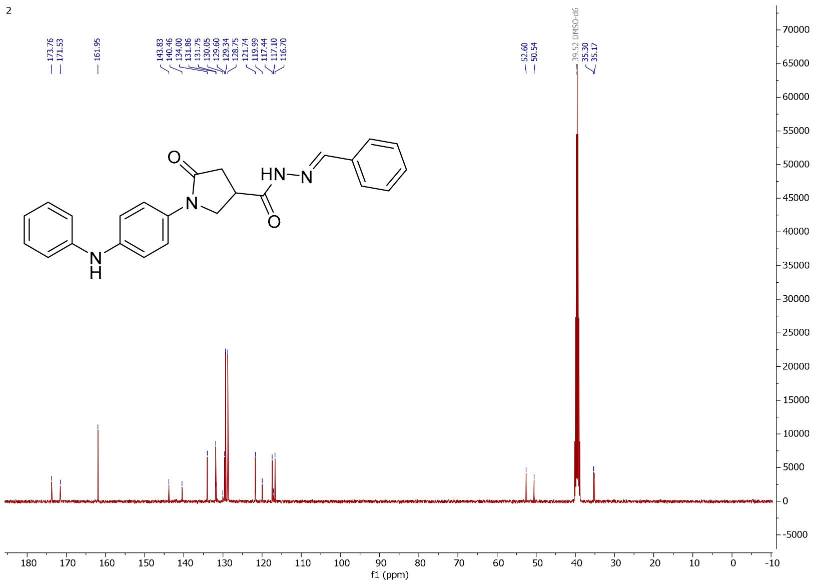

Figure S2. ^{13}C NMR (101 MHz, DMSO-d6) spectrum of **2**

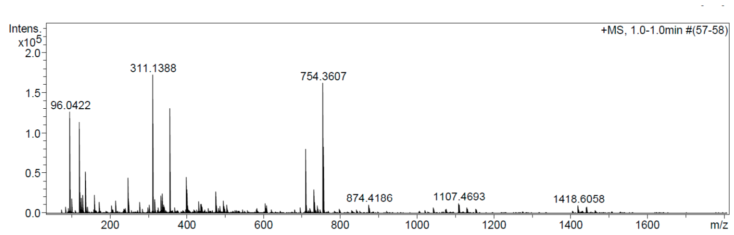

Meas. m/z	#	Formula	Score	m/z	err [ppm]	Mean err [ppm]	mSigma	rdb	e¯ Conf	N-Rule
311.1388	1	C 18 H 19 N 2 O 3	100.00	311.1390	0.8	1.1	10.4	10.5	even	ok
356.1969	1	C 20 H 26 N 3 O 3	100.00	356.1969	-0.2	-0.1	3.1	9.5	even	ok
399.1708	1	C 25 H 23 N 2 O 3	100.00	399.1703	-1.2	-1.8	18.7	15.5	even	ok
709.3025	1	C 43 H 41 N 4 O 6	100.00	709.3021	-0.7	-0.6	5.0	25.5	even	ok
	2	C 40 H 33 N 14	40.35	709.3007	-2.6	-2.5	10.4	31.5	even	ok
	3	C 42 H 45 O 10	39.88	709.3007	-2.6	-2.4	11.4	20.5	even	ok
	4	C 44 H 37 N 8 O 2	70.06	709.3034	1.2	1.3	13.9	30.5	even	ok
754.3607	1	C 45 H 48 N 5 O 6	100.00	754.3599	-1.1	-0.9	4.5	24.5	even	ok
	2	C 46 H 44 N 9 O 2	96.12	754.3612	0.7	0.9	15.2	29.5	even	ok

Figure S3. HRMS spectrum of **2**

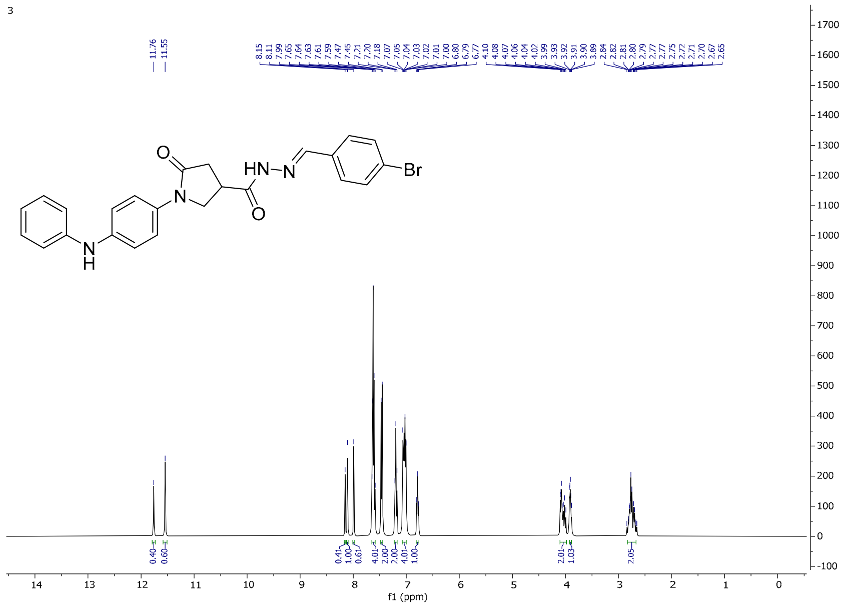

Figure S4. ^{1}H NMR (400 MHz, DMSO-d_6) spectrum of **3**

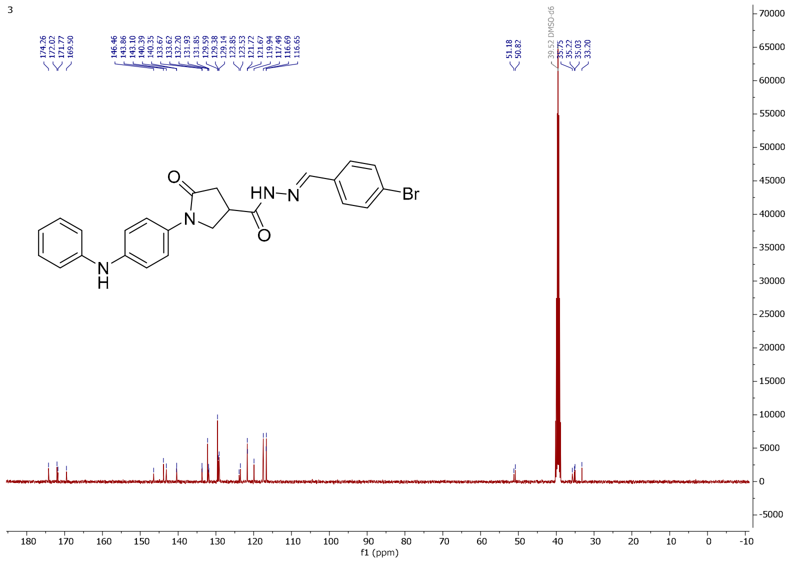

Figure S5. ^{13}C NMR (101 MHz, DMSO-d_6) spectrum of **3**

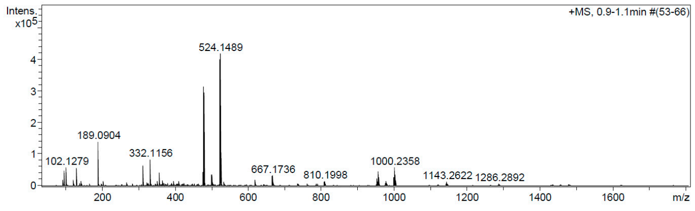

Meas. m/z	#	Formula	Score	m/z	err [ppm]	Mean err [ppm]	mSigma	rdb	e⁻ Conf	N-Rule
477.0926	1	C 21 H 9 N 12 O 3	80.90	477.0915	-2.3	-1.9	25.7	23.5	even	ok
	2	C 23 H 26 Br O 6	47.24	477.0907	-3.9	-3.7	26.6	10.5	even	ok
	3	C 24 H 22 Br N 4 O 2	100.00	477.0921	-1.1	-0.9	29.4	15.5	even	ok
	4	C 25 H 13 N 6 O 5	44.32	477.0942	3.3	3.7	37.2	22.5	even	ok
	5	C 36 H 13 O 2	11.33	477.0910	-3.3	-2.9	78.4	30.5	even	ok
	6	C 24 H 17 N 2 O 9	3.23	477.0929	0.5	3.1	120.7	17.5	even	ok
522.1506	1	C 26 H 29 Br N 5 O 2	100.00	522.1499	-1.4	-1.2	23.9	14.5	even	ok
	2	C 23 H 16 N 13 O 3	61.95	522.1494	-2.4	-2.3	30.7	22.5	even	ok
	3	C 27 H 20 N 7 O 5	48.31	522.1520	2.7	2.9	37.2	21.5	even	ok
	4	C 38 H 20 N O 2	7.78	522.1489	-3.4	-3.2	83.5	29.5	even	ok
	5	C 26 H 24 N 3 O 9	1.66	522.1507	0.1	2.6	133.1	16.5	even	ok

Figure S6. HRMS spectrum of **3**

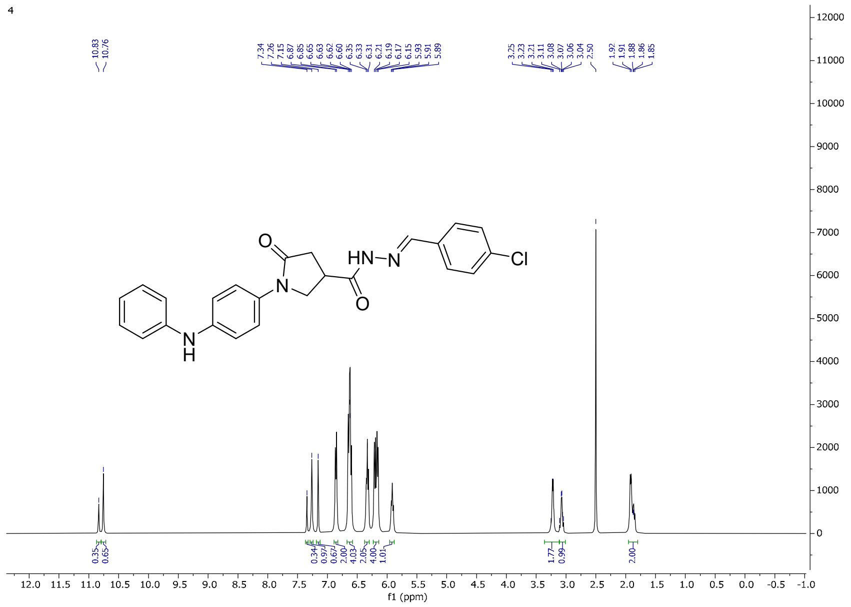

Figure S7. ^{1}H NMR (400 MHz, DMSO-d_6) spectrum of **4**

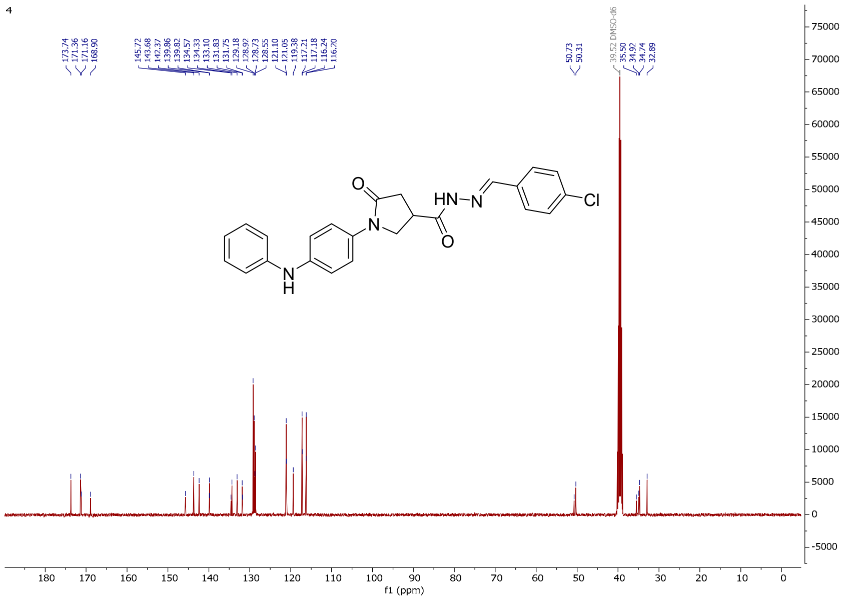

Figure S8. ^{13}C NMR (101 MHz, DMSO-d_6) spectrum of **4**

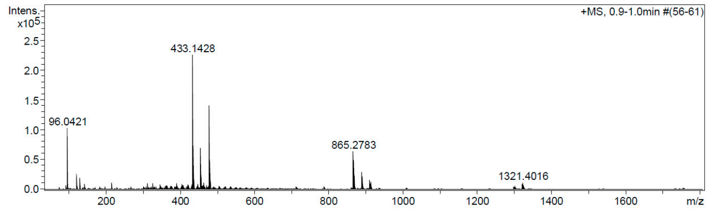

Meas. m/z	#	Formula	Score	m/z	err [ppm]	Mean err [ppm]	mSigma	rdb	e⁻ Conf	N-Rule
433.1428	1	C 23 H 26 Cl O 6	49.57	433.1412	-3.5	-3.2	17.9	10.5	even	ok
	2	C 24 H 22 Cl N 4 O 2	100.00	433.1426	-0.4	-0.2	19.3	15.5	even	ok
	3	C 29 H 21 O 4	47.67	433.1434	1.5	1.9	40.4	19.5	even	ok
455.1245	1	C 21 H 20 Cl N 6 O 4	53.09	455.1229	-3.5	-3.6	8.8	14.5	even	ok
	2	C 22 H 16 Cl N 10	100.00	455.1242	-0.6	-0.7	14.7	19.5	even	ok
	3	C 25 H 24 Cl O 6	62.12	455.1256	2.4	2.4	16.9	13.5	even	ok
	4	C 26 H 19 N 2 O 6	45.77	455.1238	-1.6	-1.5	38.3	18.5	even	ok
	5	C 27 H 15 N 6 O 2	44.12	455.1251	1.3	1.4	42.1	23.5	even	ok
478.2006	1	C 26 H 29 Cl N 5 O 2	100.00	478.2004	-0.4	-0.3	13.0	14.5	even	ok
	2	C 25 H 33 Cl N O 6	44.47	478.1991	-3.2	-3.1	17.2	9.5	even	ok
	3	C 31 H 28 N O 4	44.42	478.2013	1.4	1.7	37.9	18.5	even	ok
865.2783	1	C 54 H 38 Cl N 8 O 2	49.62	865.2801	2.0	2.3	135.9	39.5	even	ok
	2	C 53 H 42 Cl N 4 O 6	100.00	865.2787	0.5	0.8	137.3	34.5	even	ok
	3	C 52 H 46 Cl O 10	72.38	865.2774	-1.1	-0.7	138.5	29.5	even	ok
	[illegible]	[illegible]	[illegible]	[illegible]	[illegible]	[illegible]	[illegible]	[illegible]	[illegible]	[illegible]

Figure S9. HRMS spectrum of **4**

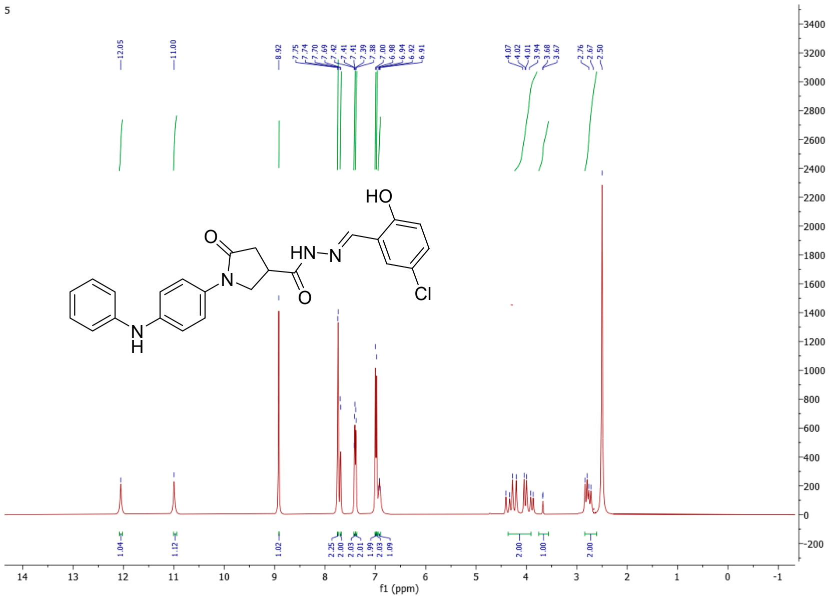

Figure S10. ^{1}H NMR (400 MHz, DMSO-d_6) spectrum of **5**

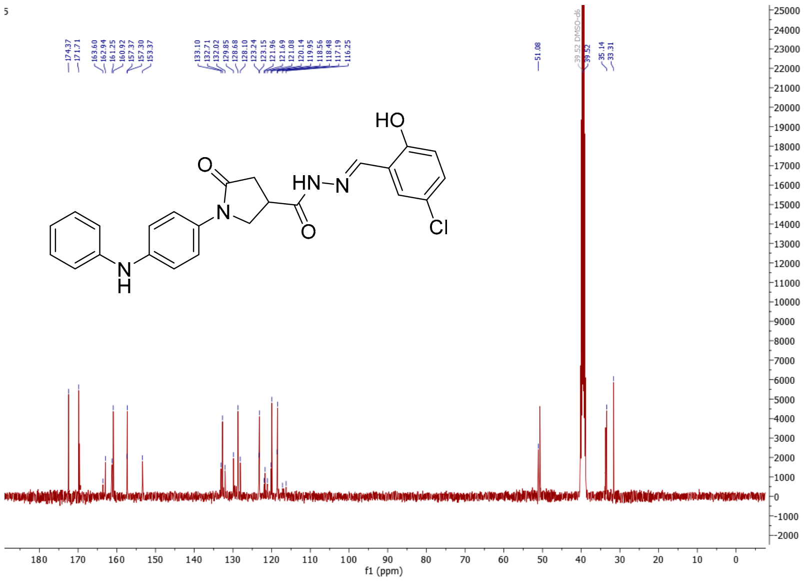

Figure S11. ^{13}C NMR (101 MHz, DMSO-d_6) spectrum of **5**

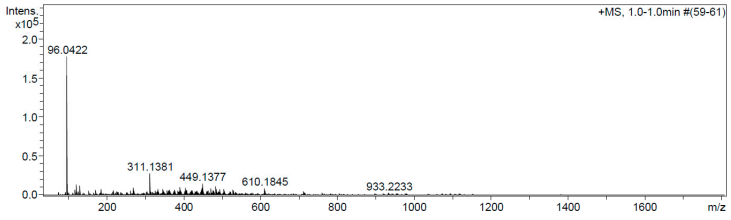

Meas. m/z	#	Formula	Score	m/z	err [ppm]	Mean err [ppm]	mSigma	rdb	e⁻ Conf	N-Rule
311.1381	1	C 14 H 15 N 8 O	59.68	311.1363	-5.6	-5.6	14.5	11.5	even	ok
	2	C 18 H 19 N 2 O 3	100.00	311.1390	3.0	3.1	14.7	10.5	even	ok
	3	C 13 H 20 Cl N 6 O	0.14	311.1382	0.2	0.3	169.1	6.5	even	ok
	4	C 12 H 24 Cl N 2 O 5	0.05	311.1368	-4.0	-3.9	173.8	1.5	even	ok
449.1377	1	C 24 H 22 Cl N 4 O 3	100.00	449.1375	-0.5	-0.7	25.5	15.5	even	ok
	2	C 23 H 26 Cl O 7	39.64	449.1362	-3.5	-3.6	33.3	10.5	even	ok
	3	C 29 H 21 O 5	1.19	449.1384	1.4	5.3	130.8	19.5	even	ok
	4	C 30 H 17 N 4 O	0.44	449.1397	4.4	8.3	133.4	24.5	even	ok

Figure S12. HRMS spectrum of **5**

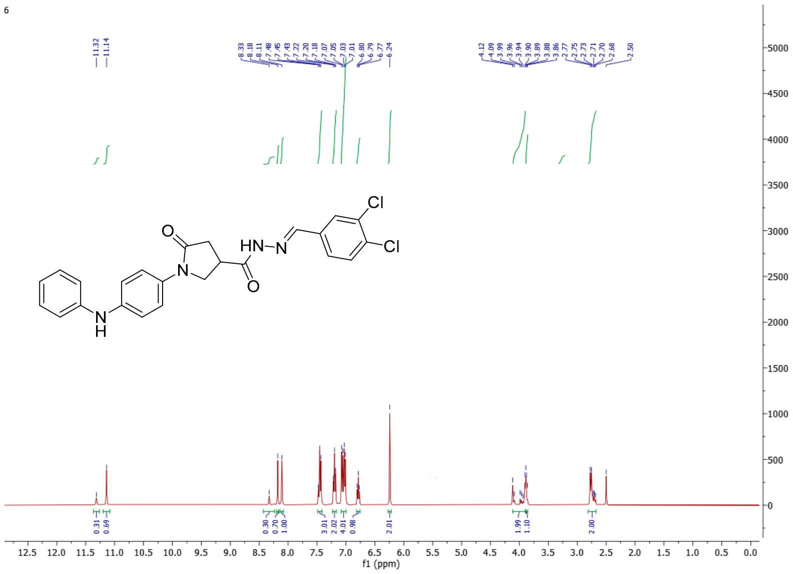

Figure S13. ^{1}H NMR (400 MHz, DMSO-d_6) spectrum of **6**

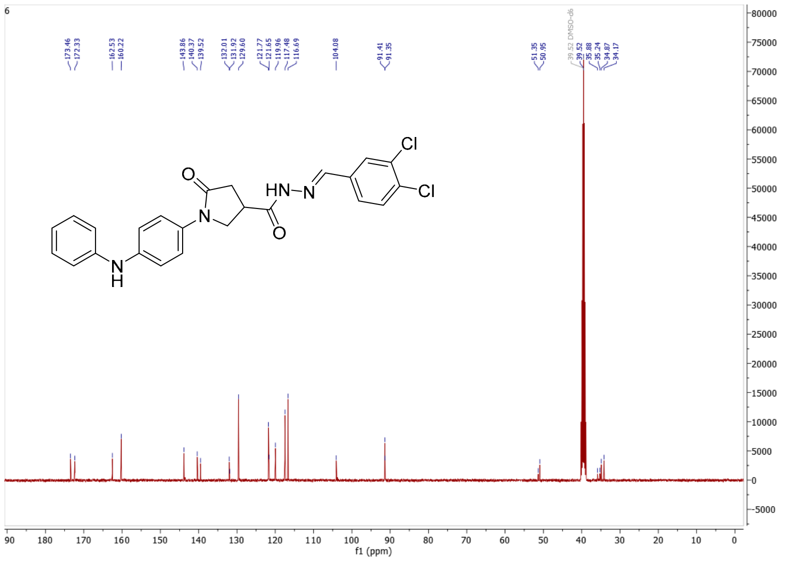

Figure S14. ^{13}C NMR (400 MHz, DMSO-d_6) spectrum of **6**

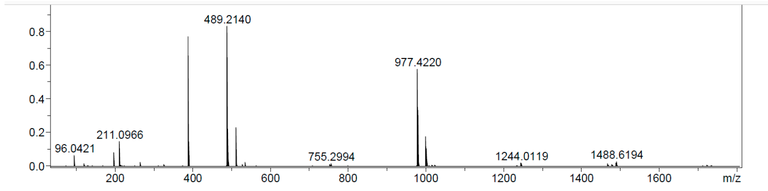

Meas. m/z	#	Formula	Score	m/z	err [ppm]	Mean err [ppm]	mSigma	rdb	e⁻ Conf	N-Rule
389.1711	1	C 20 H 25 N 2 O 6	100.00	389.1707	-1.0	-0.7	18.3	9.5	even	ok
	2	C 17 H 17 N 12	43.29	389.1694	-4.5	-4.3	20.7	15.5	even	ok
	3	C 21 H 21 N 6 O 2	54.20	389.1721	2.4	2.7	32.8	14.5	even	ok
	4	C 16 H 22 Cl N 10	0.30	389.1712	0.2	0.4	155.3	10.5	even	ok
	5	C 15 H 26 Cl N 6 O 4	0.16	389.1699	-3.2	-3.1	156.2	5.5	even	ok
	6	C 19 H 30 Cl O 6	0.10	389.1725	3.7	3.9	160.5	4.5	even	ok
	7	C 15 H 27 Cl 2 N 8	0.00	389.1730	4.9	5.1	276.6	5.5	even	ok
	8	C 14 H 31 Cl 2 N 4 O 4	0.00	389.1717	1.5	1.7	277.9	0.5	even	ok
489.2140	1	C 27 H 29 N 4 O 5	100.00	489.2132	-1.6	-1.3	21.3	15.5	even	ok
	2	C 28 H 25 N 8 O	82.93	489.2146	1.2	1.4	33.5	20.5	even	ok
	3	C 22 H 30 Cl N 8 O 3	0.30	489.2124	-3.3	-3.8	146.3	11.5	even	ok
	4	C 26 H 34 Cl N 2 O 5	0.29	489.2151	2.2	1.8	152.7	10.5	even	ok
	5	C 21 H 35 Cl 2 N 6 O 3	0.00	489.2142	0.4	0.0	271.4	6.5	even	ok
	6	C 20 H 39 Cl 2 N 2 O 7	0.00	489.2129	-2.3	-2.7	271.5	1.5	even	ok

Figure S15. HRMS spectrum of **6**

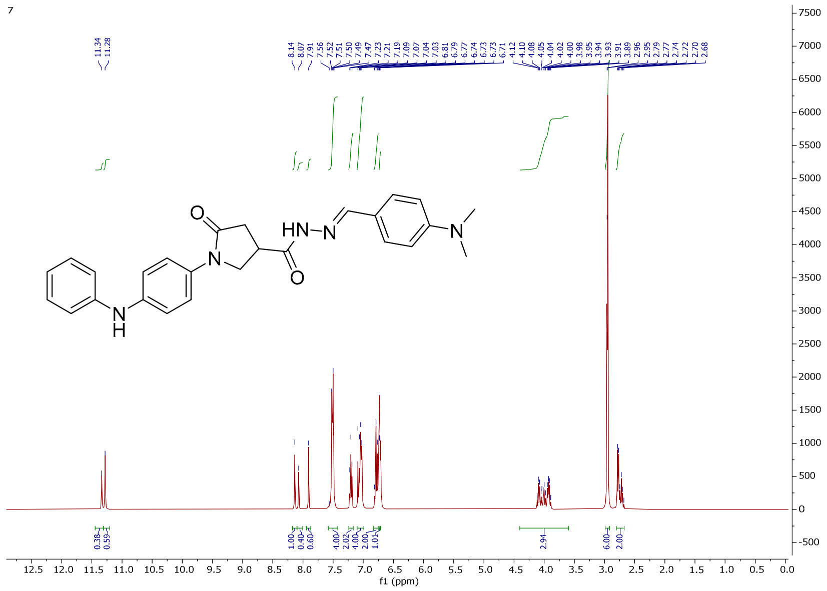

Figure S16. ^{1}H NMR (400 MHz, DMSO-d_6) spectrum of **7**

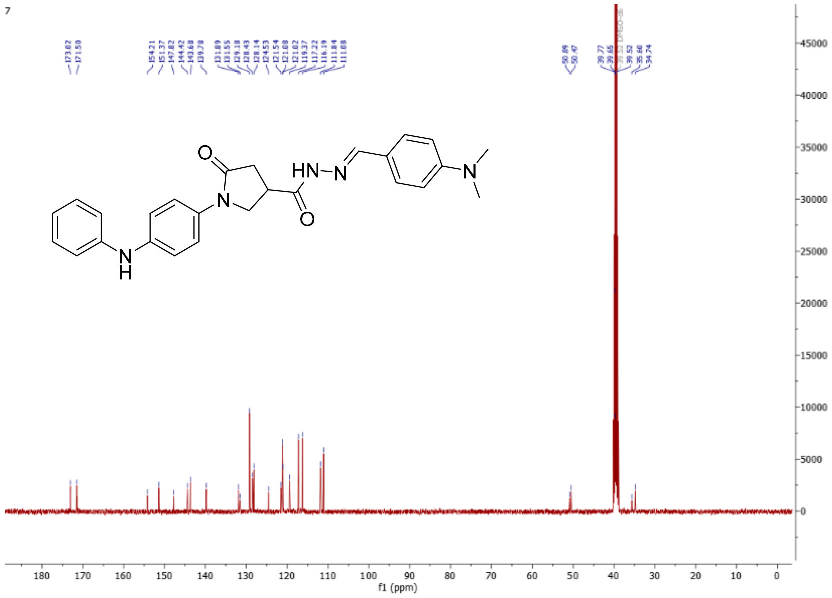

Figure S17. ^{13}C NMR (101 MHz, DMSO-d_6) spectrum of **7**

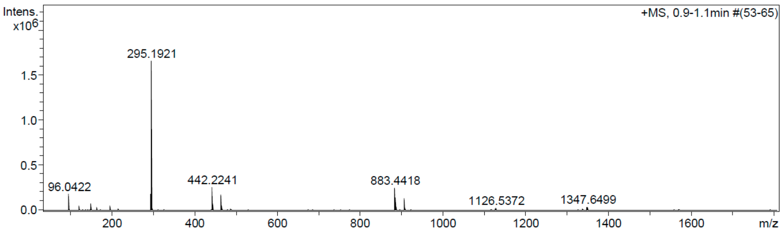

Meas. m/z	#	Formula	Score	m/z	err [ppm]	Mean err [ppm]	mSigma	rdb	e⁻ Conf	N-Rule
295.1921	1	C 17 H 27 O 4	64.04	295.1904	-5.9	-5.6	21.1	4.5	even	ok
	2	C 18 H 23 N 4	100.00	295.1917	-1.4	-1.1	35.2	9.5	even	ok
442.2241	1	C 25 H 32 N O 6	52.90	442.2224	-3.9	-3.6	4.2	10.5	even	ok
	2	C 26 H 28 N 5 O 2	100.00	442.2238	-0.8	-0.6	11.8	15.5	even	ok
464.2061	1	C 27 H 30 N O 6	100.00	464.2068	1.5	1.8	9.4	13.5	even	ok
	2	C 24 H 22 N 11	98.28	464.2054	-1.4	-1.2	10.7	19.5	even	ok

Figure S18. HRMS spectrum of **7**

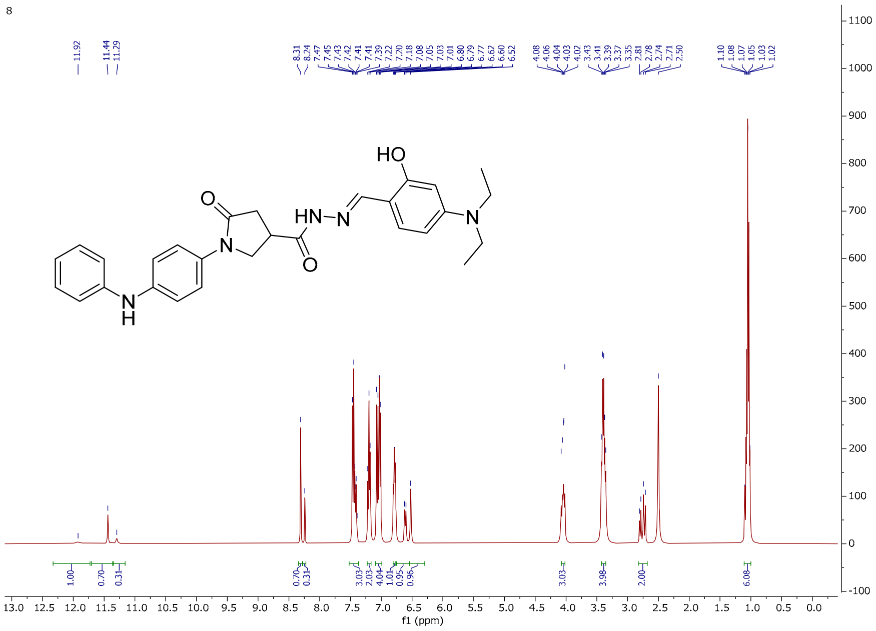

Figure S19. ^{1}H NMR (400 MHz, DMSO-d_6) spectrum of **8**

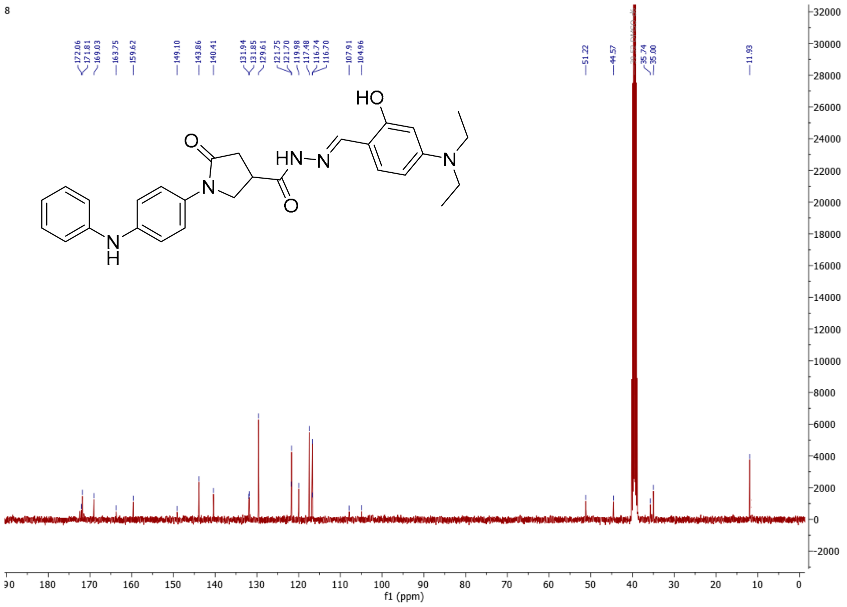

Figure S20. ^{13}C NMR (101 MHz, DMSO-d_6) spectrum of **8**

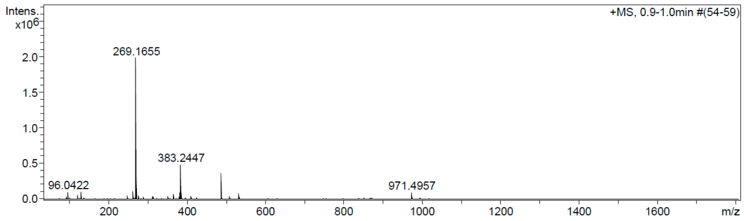

Meas. m/z	#	Formula	Score	m/z	err [ppm]	Mean err [ppm]	mSigma	rdb	e⁻ Conf	N-Rule
269.1655	1	C 17 H 21 N 2 O	100.00	269.1648	-2.5	-2.3	33.8	8.5	even	ok
383.2447	1	C 21 H 35 O 6	57.66	383.2428	-4.9	-4.6	9.7	4.5	even	ok
	2	C 22 H 31 N 4 O 2	100.00	383.2442	-1.4	-1.1	22.7	9.5	even	ok
486.2510	1	C 28 H 32 N 5 O 3	100.00	486.2500	-2.1	-1.8	10.6	15.5	even	ok
971.4957	1	C 59 H 71 O 12	54.80	971.4940	-1.8	-1.5	11.4	24.5	even	ok
	2	C 57 H 59 N 14 O 2	45.82	971.4940	-1.8	-1.6	19.9	35.5	even	ok
	3	C 60 H 67 N 4 O 8	100.00	971.4953	-0.4	-0.1	19.9	29.5	even	ok
	4	C 61 H 63 N 8 O 4	56.08	971.4967	1.0	1.2	31.5	34.5	even	ok
	5	C 48 H 71 N 6 O 15	33.65	971.4972	1.5	1.8	39.0	16.5	even	ok

Figure S21. HRMS spectrum of **8**

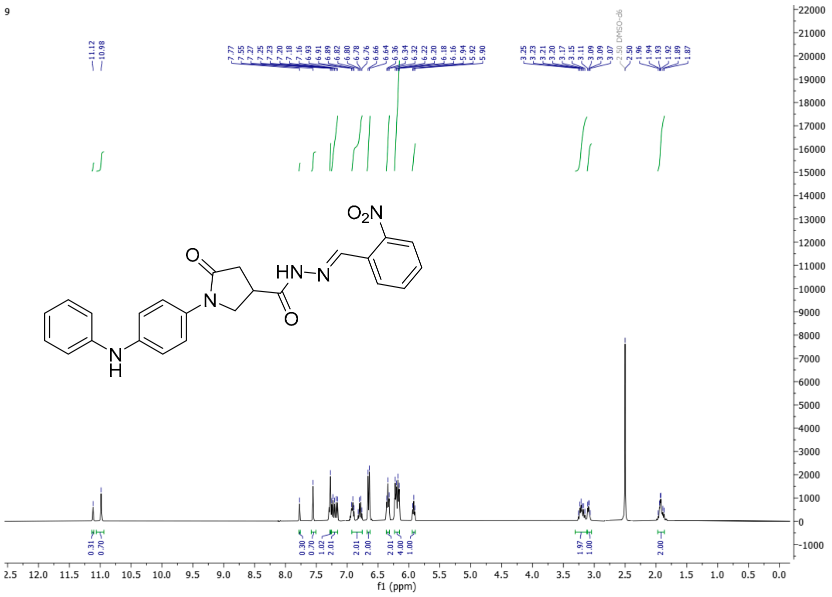

Figure S22. ^{1}H NMR (400 MHz, DMSO-d$_6$) spectrum of **9**

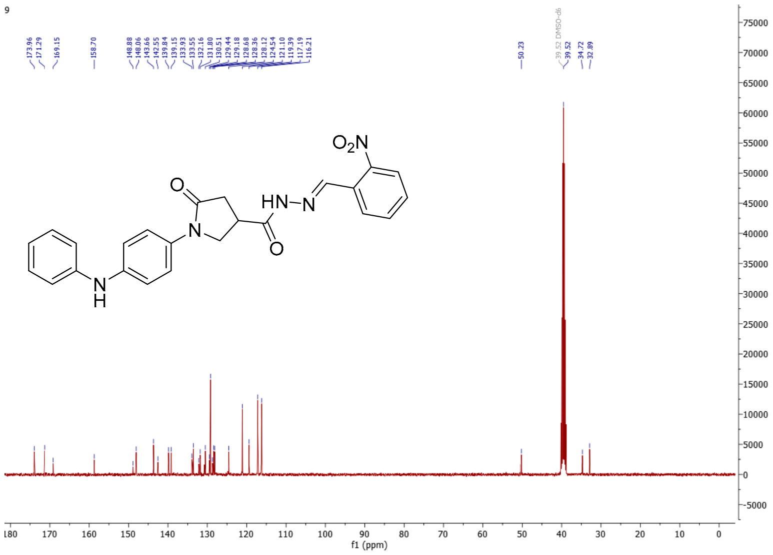

Figure S23. ^{13}C NMR (101 MHz, DMSO-d$_6$) spectrum of **9**

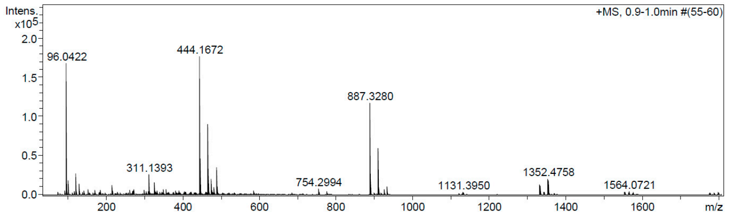

Meas. m/z	#	Formula	Score	m/z	err [ppm]	Mean err [ppm]	mSigma	rdb	e⁻ Conf	N-Rule
444.1672	1	C 23 H 26 N O 8	45.16	444.1653	-4.4	-4.0	6.0	11.5	even	ok
	2	C 24 H 22 N 5 O 4	100.00	444.1666	-1.4	-1.0	8.1	16.5	even	ok
	3	C 25 H 18 N 9	69.63	444.1680	1.6	2.0	22.9	21.5	even	ok
466.1491	1	C 22 H 16 N 11 O 2	91.53	466.1483	-1.7	-1.4	3.4	20.5	even	ok
	2	C 25 H 24 N O 8	100.00	466.1496	1.2	1.6	4.6	14.5	even	ok
	3	C 26 H 20 N 5 O 4	35.95	466.1510	4.1	4.4	15.4	19.5	even	ok
887.3280	1	C 51 H 51 O 14	100.00	887.3273	-0.8	-0.4	16.7	26.5	even	ok
	2	C 52 H 47 N 4 O 10	79.22	887.3287	0.8	1.1	27.3	31.5	even	ok
	3	C 49 H 39 N 14 O 4	76.27	887.3273	-0.8	-0.5	28.7	37.5	even	ok

Figure S24. HRMS spectrum of **9**

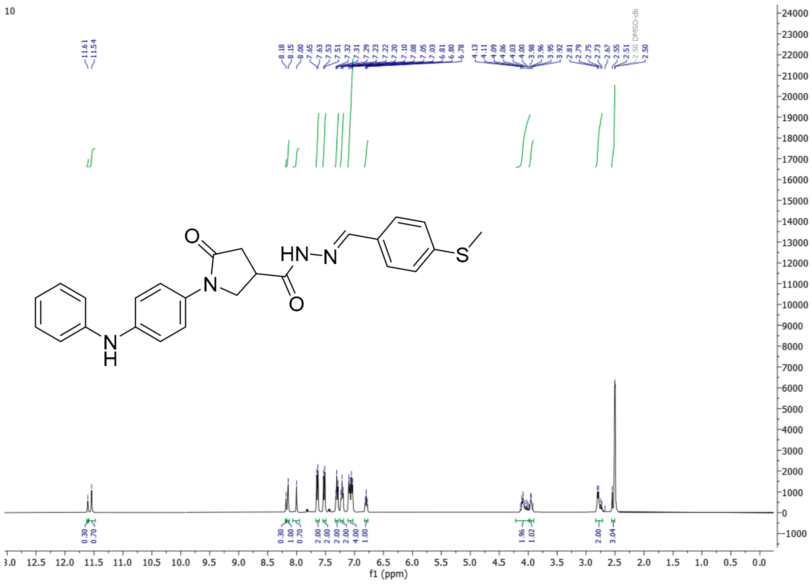

Figure S25. ^{1}H NMR (400 MHz, DMSO-d_6) spectrum of **10**

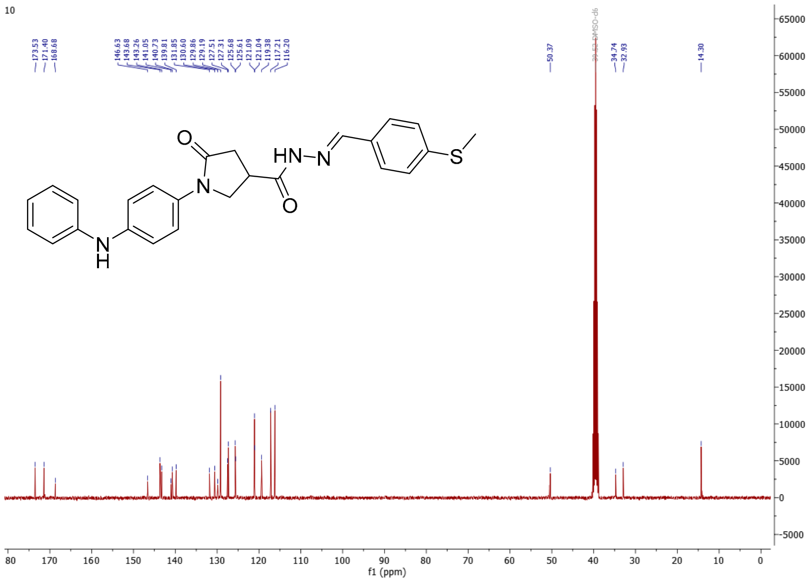

Figure S26. ^{13}C NMR (101 MHz, DMSO-d_6) spectrum of **10**

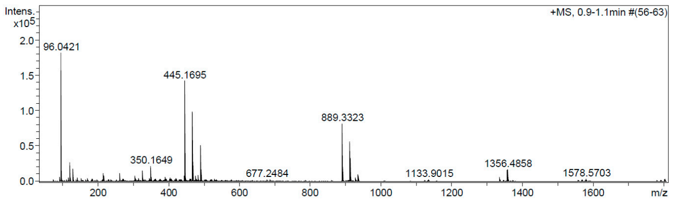

Meas. m/z	#	Formula	Score	m/z	err [ppm]	Mean err [ppm]	mSigma	rdb	e⁻ Conf	N-Rule
445.1695	1	C 25 H 25 N 4 O 2 S	100.00	445.1693	-0.5	-0.8	12.0	15.5	even	ok
	2	C 24 H 29 O 6 S	40.94	445.1679	-3.5	-3.8	19.5	10.5	even	ok
	3	C 33 H 21 N 2	56.98	445.1699	0.9	1.9	33.5	24.5	even	ok
	4	C 20 H 29 O 11	36.41	445.1704	2.1	3.0	40.5	6.5	even	ok
467.1515	1	C 23 H 19 N 10 S	100.00	467.1509	-1.2	-1.6	11.6	19.5	even	ok
	2	C 22 H 23 N 6 O 4 S	43.67	467.1496	-4.1	-4.4	11.9	14.5	even	ok
	3	C 26 H 27 O 6 S	82.28	467.1523	1.7	1.4	15.9	13.5	even	ok
	4	C 19 H 19 N 10 O 5	30.38	467.1534	4.1	5.0	27.7	15.5	even	ok
	5	C 30 H 19 N 4 O 2	45.96	467.1503	-2.7	-1.6	30.0	23.5	even	ok
490.2273	1	C 27 H 32 N 5 O 2 S	100.00	490.2271	-0.4	-0.9	14.8	14.5	even	ok
	2	C 26 H 36 N O 6 S	40.85	490.2258	-3.2	-3.6	22.4	9.5	even	ok
	3	C 35 H 28 N 3	67.07	490.2278	0.9	1.7	28.5	23.5	even	ok
889.3323	1	C 57 H 49 N 2 O 6 S	58.77	889.3306	-1.9	-0.9	12.3	34.5	even	ok
	2	C 58 H 45 N 6 O 2 S	100.00	889.3319	-0.4	0.6	23.7	39.5	even	ok

Figure S27. HRMS spectrum of **10**

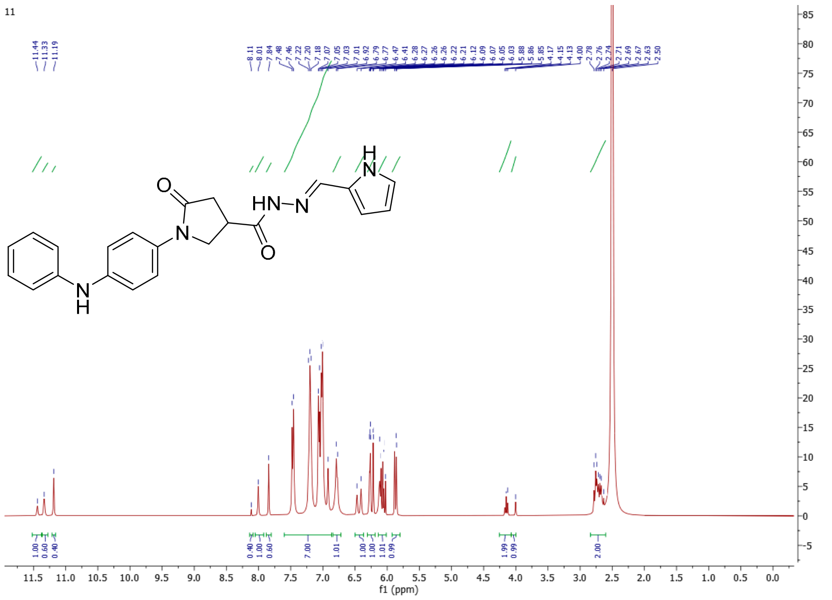

Figure S28. ^{1}H NMR (400 MHz, DMSO-d$_6$) spectrum of **11**

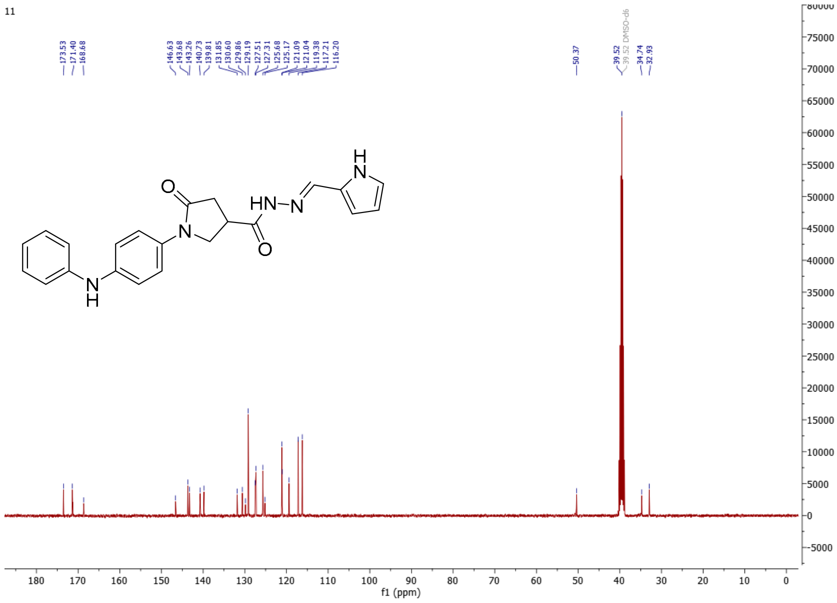

Figure S29. ^{13}C NMR (101 MHz, DMSO-d$_6$) spectrum of **11**

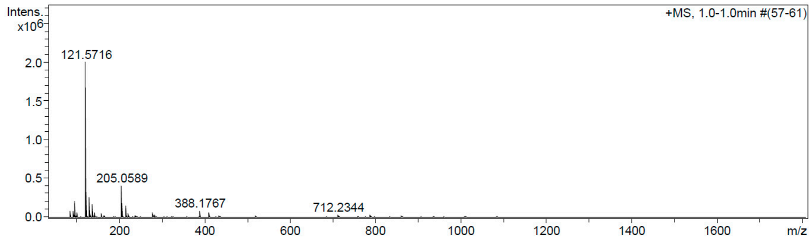

Meas. m/z	#	Formula	Score	m/z	err [ppm]	Mean err [ppm]	mSigma	rdb	e⁻ Conf	N-Rule
388.1767	1	C 22 H 22 N 5 O 2	100.00	388.1768	0.3	0.8	4.6	14.5	even	ok
	2	C 21 H 26 N O 6	50.91	388.1755	-3.1	-2.7	10.9	9.5	even	ok

Figure S30. HRMS spectrum of **11**

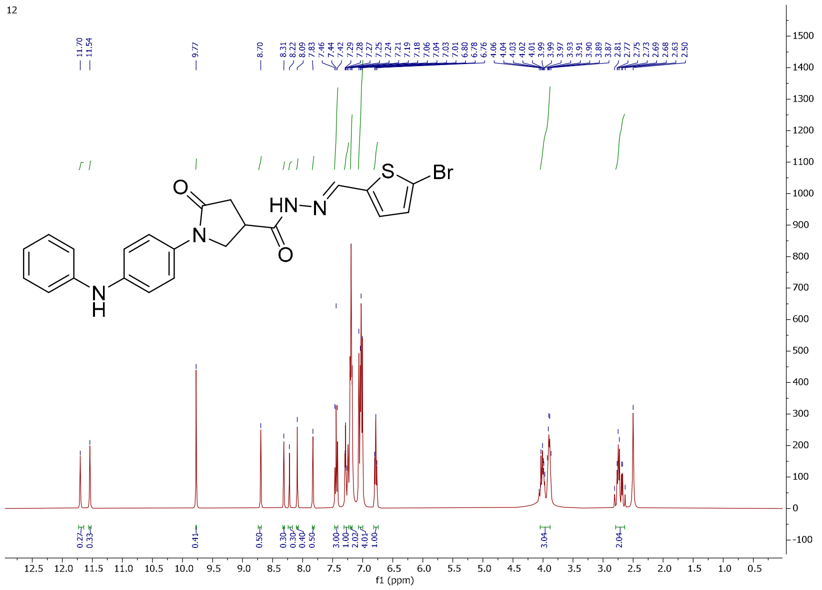

Figure S31. ^{1}H NMR (400 MHz, DMSO-d$_6$) spectrum of **12**

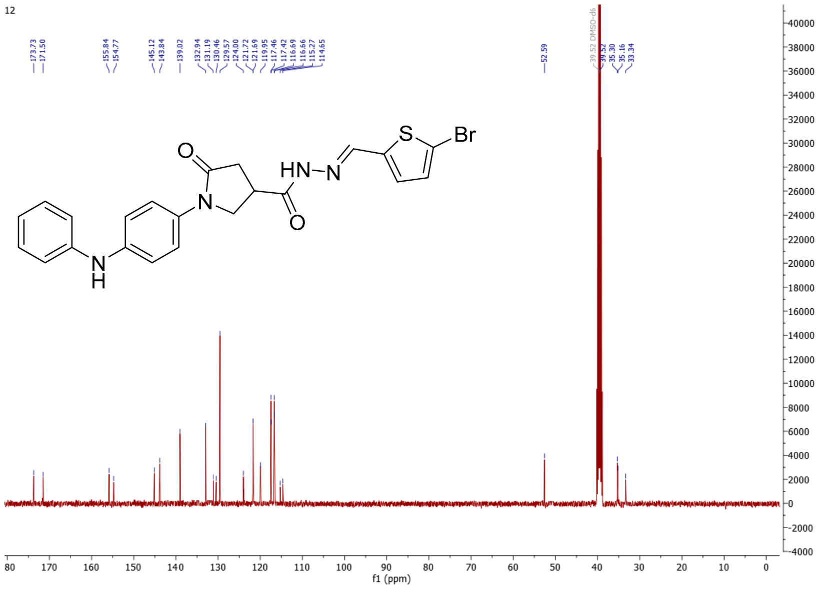

Figure S32. ^{13}C NMR (101 MHz, DMSO-d$_6$) spectrum of **12**

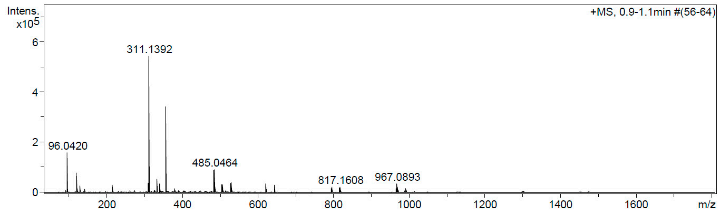

Meas. m/z	#	Formula	Score	m/z	err [ppm]	Mean err [ppm]	mSigma	rdb	e⁻ Conf	N-Rule
311.1392	1	C 18 H 19 N 2 O 3	100.00	311.1390	-0.5	-0.2	18.4	10.5	even	ok
	2	C 11 H 19 N 8 O S	65.47	311.1397	1.7	1.9	29.2	6.5	even	ok
	3	C 10 H 23 N 4 O 5 S	46.59	311.1384	-2.6	-2.4	37.5	1.5	even	ok
	4	C 4 H 11 N 18	11.68	311.1409	5.4	5.1	64.4	8.5	even	ok
356.1970	1	C 20 H 26 N 3 O 3	100.00	356.1969	-0.5	-0.1	15.7	9.5	even	ok
	2	C 13 H 26 N 9 O S	59.27	356.1976	1.5	1.7	31.9	5.5	even	ok
	3	C 6 H 18 N 19	24.33	356.1987	4.7	1.9	39.9	7.5	even	ok
	4	C 12 H 30 N 5 O 5 S	40.80	356.1962	-2.3	-2.1	40.3	0.5	even	ok
483.0483	1	C 22 H 20 Br N 4 O 2 S	100.00	483.0485	0.5	0.5	19.7	14.5	even	ok
	2	C 21 H 24 Br O 6 S	54.56	483.0471	-2.3	-2.2	26.4	9.5	even	ok
	3	C 15 H 12 Br N 14 O	39.01	483.0496	2.9	3.4	33.5	16.5	even	ok
	4	C 30 H 16 Br N 2	44.32	483.0491	1.8	2.6	40.0	23.5	even	ok
	5	C 17 H 24 Br O 11	31.39	483.0497	2.9	3.5	41.5	5.5	even	ok
	6	C 26 H 7 N 6 O 5	38.31	483.0472	-2.1	-1.5	42.4	26.5	even	ok
	7	[illegible]	[illegible]	[illegible]	[illegible]	[illegible]	[illegible]	[illegible]	[illegible]	[illegible]

Figure S33. HRMS spectrum of **12**

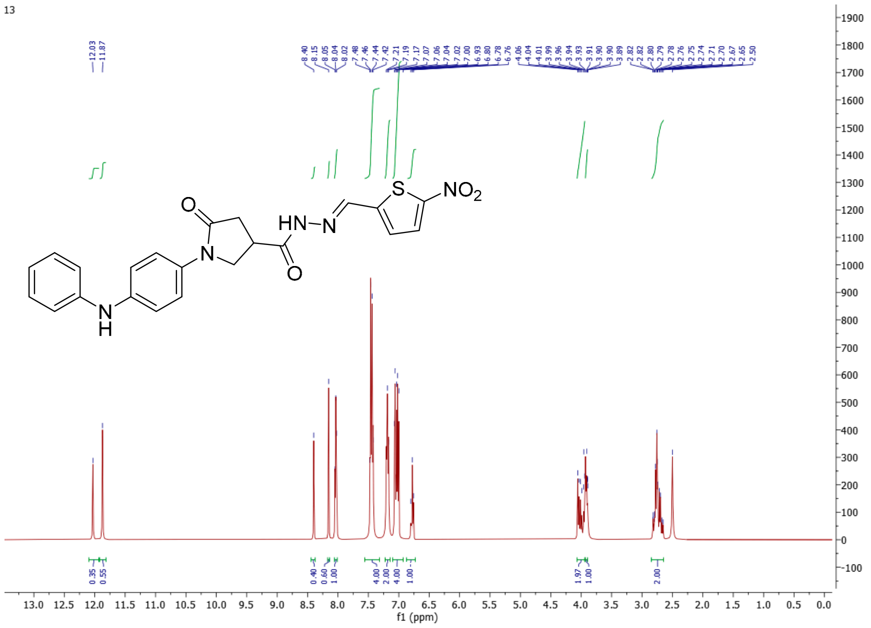

Figure S34. ^{1}H NMR (400 MHz, DMSO-d$_6$) spectrum of **13**

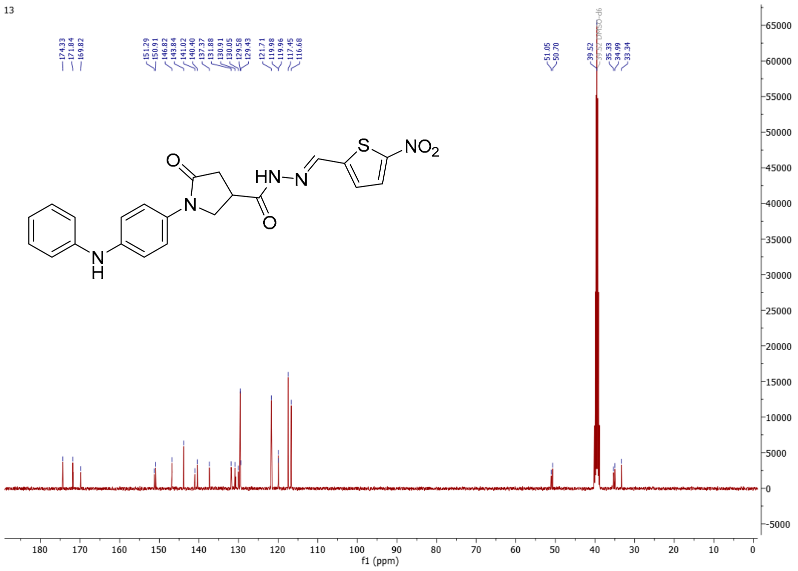

Figure S35. ^{13}C NMR (101 MHz, DMSO-d$_6$) spectrum of **13**

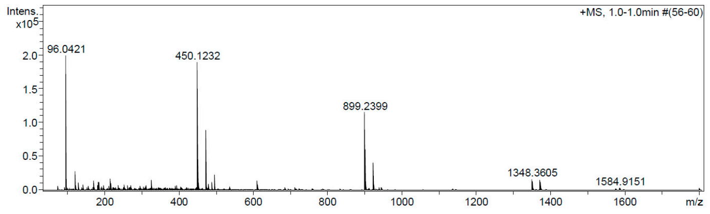

Meas. m/z	#	Formula	Score	m/z	err [ppm]	Mean err [ppm]	mSigma	rdb	e⁻ Conf	N-Rule
450.1232	1	C 21 H 24 N O 8 S	55.48	450.1217	-3.3	-3.4	15.4	10.5	even	ok
	2	C 15 H 12 N 15 O 3	66.15	450.1242	2.3	3.1	20.0	17.5	even	ok
	3	C 17 H 24 N O 13	63.81	450.1242	2.3	3.1	21.3	6.5	even	ok
	4	C 22 H 20 N 5 O 4 S	100.00	450.1231	-0.3	-0.5	21.5	15.5	even	ok
	5	C 14 H 16 N 11 O 7	79.55	450.1229	-0.7	-0.1	28.0	12.5	even	ok
	6	C 8 H 12 N 21 O S	33.81	450.1249	3.8	3.4	30.1	13.5	even	ok
	7	C 23 H 16 N 9 S	45.02	450.1244	2.7	2.4	31.9	20.5	even	ok
	8	C 11 H 8 N 21 O	33.31	450.1215	-3.7	-3.5	32.3	18.5	even	ok
	9	C 13 H 20 N 7 O 11	29.14	450.1215	-3.7	-3.0	37.7	7.5	even	ok
	10	C 10 H 24 N 7 O 11 S	23.36	450.1249	3.8	3.6	43.9	2.5	even	ok
	11	C 7 H 16 N 17 O 5 S	43.54	450.1236	0.8	0.5	49.3	8.5	even	ok
	12	C 30 H 16 N 3 O 2	37.45	450.1237	1.2	2.3	51.9	24.5	even	ok
899.2399	1	C 40 H 39 N 10 O 13 S	56.74	899.2413	1.6	2.2	24.3	26.5	even	ok
	2	C 38 H 27 N 24 O 3 S	56.47	899.2413	1.6	2.1	24.9	37.5	even	ok
	[illegible]	[illegible]	[illegible]	[illegible]	[illegible]	[illegible]	[illegible]	[illegible]	[illegible]	[illegible]

Figure S36. HRMS spectrum of **13**

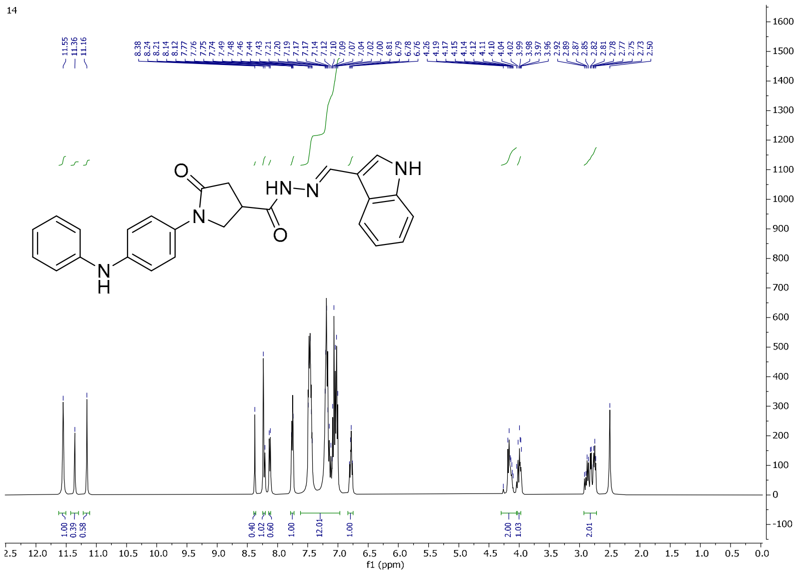

Figure S37. ^{1}H NMR (400 MHz, DMSO-d$_6$) spectrum of **14**

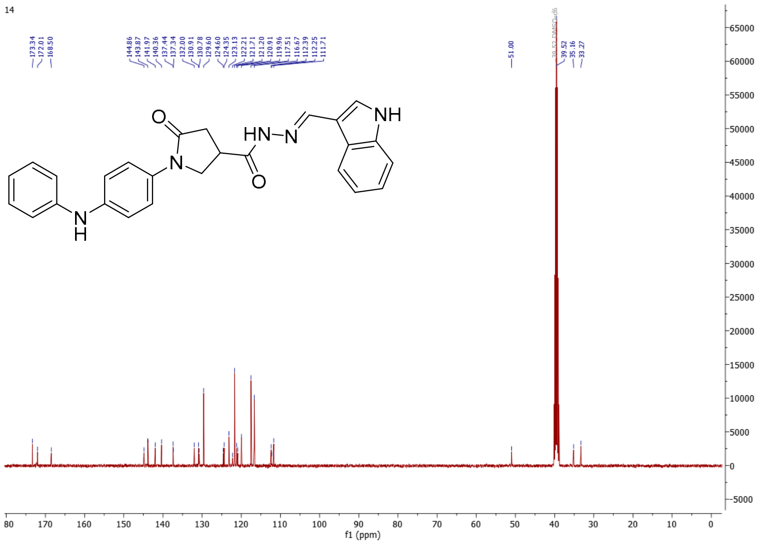

Figure S38. ^{13}C NMR (101 MHz, DMSO-d$_6$) spectrum of **14**

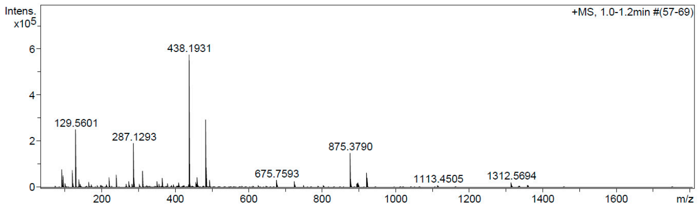

Meas. m/z	#	Formula	Score	m/z	err [ppm]	Mean err [ppm]	mSigma	rdb	e⁻ Conf	N-Rule
438.1931	1	C 25 H 28 N O 6	51.10	438.1911	-4.5	-4.2	19.5	12.5	even	ok
	2	C 26 H 24 N 5 O 2	84.91	438.1925	-1.4	-1.1	33.7	17.5	even	ok
	3	C 11 H 20 N 17 O 3	100.00	438.1930	-0.3	-0.5	36.3	10.5	even	ok
	4	C 14 H 28 N 7 O 9	54.84	438.1943	2.8	2.9	37.6	4.5	even	ok
	5	C 19 H 24 N 11 S	96.23	438.1931	0.1	0.3	38.7	13.5	even	ok
	6	C 18 H 28 N 7 O 4 S	49.61	438.1918	-2.9	-2.7	40.1	8.5	even	ok
	7	C 13 H 32 N 3 O 13	88.51	438.1930	-0.3	-0.1	41.0	-0.5	even	ok
	8	C 22 H 32 N O 6 S	39.71	438.1945	3.2	3.4	45.1	7.5	even	ok
	9	C 10 H 24 N 13 O 7	32.97	438.1916	-3.3	-3.3	50.2	5.5	even	ok
483.2509	1	C 27 H 35 N 2 O 6	54.49	483.2490	-4.0	-3.7	7.8	11.5	even	ok
	2	C 28 H 31 N 6 O 2	100.00	483.2503	-1.3	-1.0	19.4	16.5	even	ok
	3	C 21 H 31 N 12 S	73.87	483.2510	0.2	0.2	41.8	12.5	even	ok
	4	C 20 H 35 N 8 O 4 S	35.94	483.2496	-2.6	-2.6	45.7	7.5	even	ok
	5	C 24 H 39 N 2 O 6 S	30.29	483.2523	2.9	3.0	48.1	6.5	even	ok
	[illegible]	[illegible]	[illegible]	[illegible]	[illegible]	[illegible]	[illegible]	[illegible]	[illegible]	[illegible]

Figure S39. HRMS spectrum of **14**

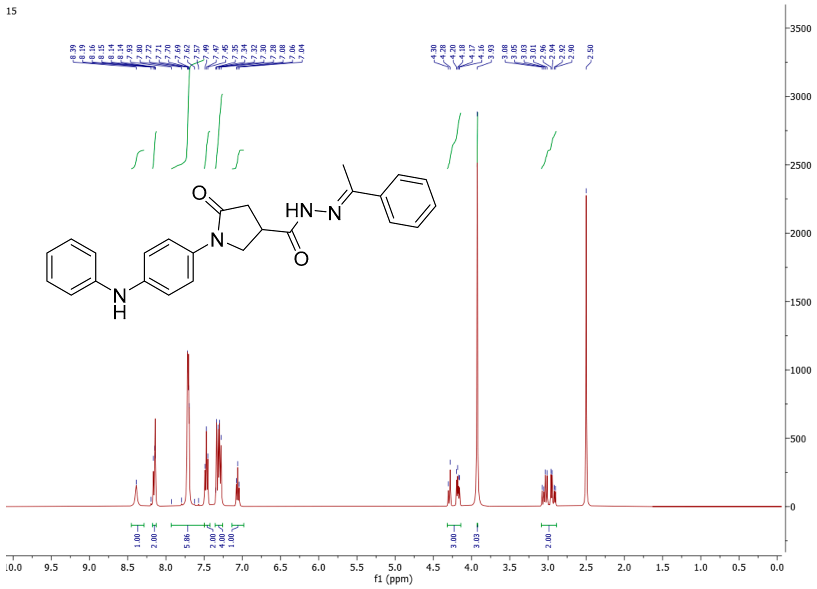

Figure S40. ^{1}H NMR (400 MHz, DMSO-d_6) spectrum of **15**

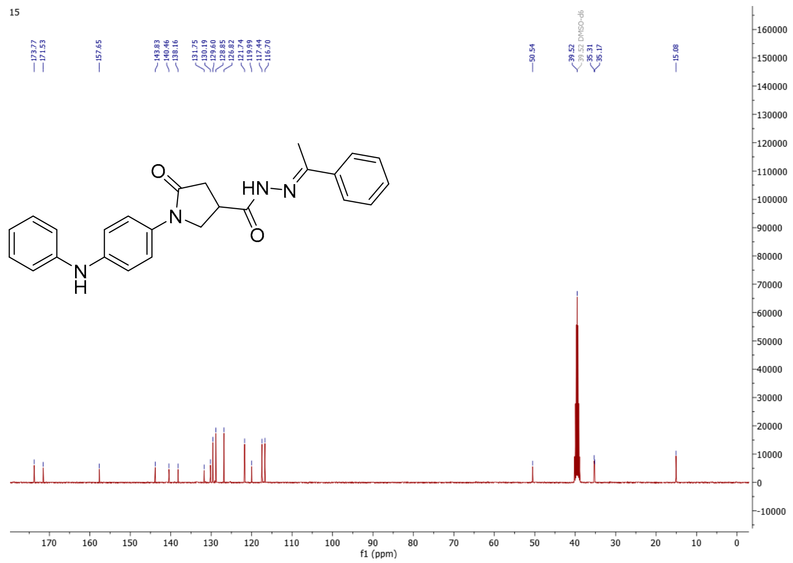

Figure S41. ^{13}C NMR (101 MHz, DMSO-d_6) spectrum of **15**

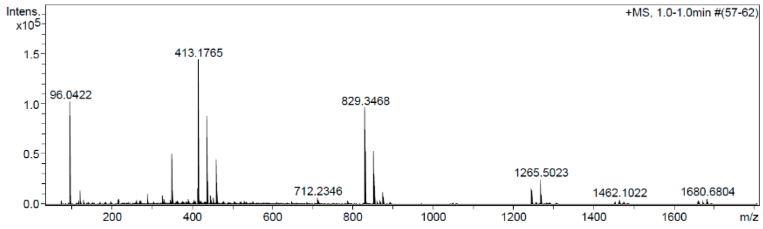

Meas. m/z	#	Formula	Score	m/z	err [ppm]	Mean err [ppm]	mSigma	rdb	e⁻ Conf	N-Rule
413.1765	1	C 25 H 24 N 4 O 2	100.00	415.1765	-0.1	0.0	5.8	15.5	even	ok
	2	C 23 H 27 O 7	47.79	415.1751	-3.4	-3.1	8.1	10.5	even	ok
437.1586	1	C 25 H 25 O 7	89.38	437.1595	2.0	2.2	2.9	13.5	even	ok
	2	C 22 H 17 N 10 O	100.00	437.1581	-1.1	-0.9	7.1	19.5	even	ok
	3	C 21 H 21 N 6 O 5	41.46	437.1568	-4.2	-4.0	11.2	14.5	even	ok
460.2346	1	C 26 H 30 N 5 O 3	100.00	460.2343	-0.5	-0.4	3.4	14.5	even	ok
	2	C 25 H 34 N O 7	43.74	460.2330	-3.4	-3.3	8.3	9.5	even	ok
829.3468	1	C 48 H 45 N 8 O 6	80.22	829.3457	-1.3	-1.0	4.3	30.5	even	ok
	2	C 52 H 49 N 2 O 8	47.91	829.3483	1.9	2.3	16.3	29.5	even	ok
	[illegible]	[illegible]	[illegible]	[illegible]	[illegible]	[illegible]	[illegible]	[illegible]	[illegible]	[illegible]

Figure S42. HRMS spectrum of **15**

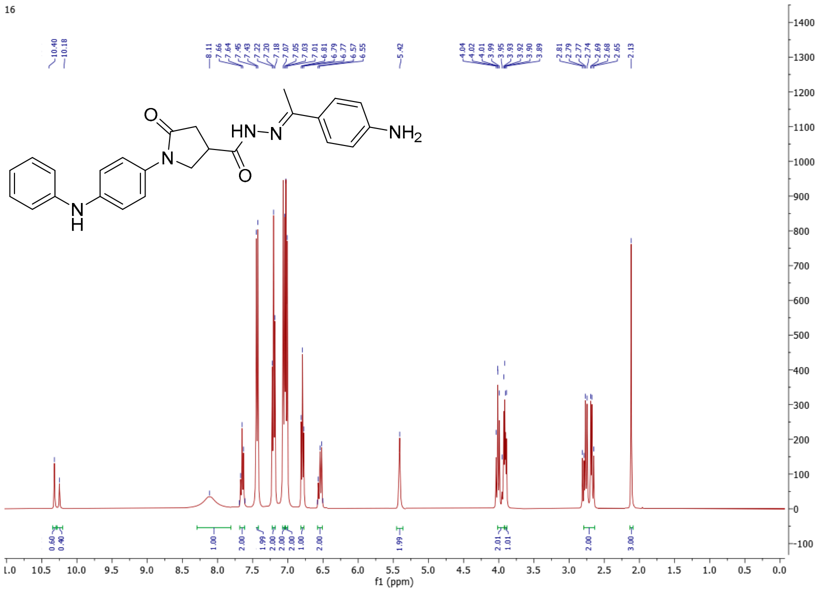

Figure S43. ^{1}H NMR (400 MHz, DMSO-d$_6$) spectrum of **16**

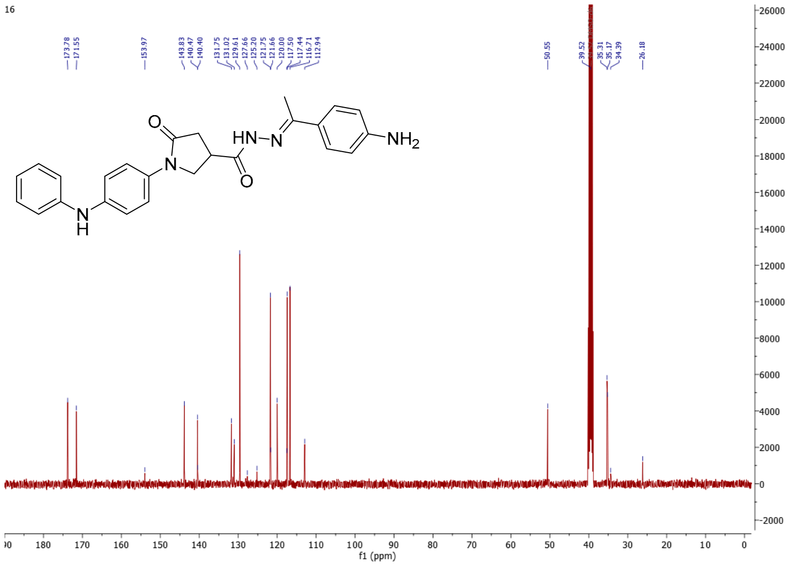

Figure S44. ^{13}C NMR (101 MHz, DMSO-d$_6$) spectrum of **16**

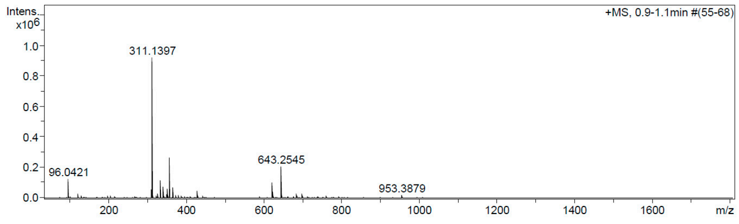

Meas. m/z	#	Formula	Score	m/z	err [ppm]	Mean err [ppm]	mSigma	rdb	e⁻ Conf	N-Rule
311.1397	1	C 18 H 19 N 2 O 3	100.00	311.1390	-2.3	-2.0	22.0	10.5	even	ok
	2	C 4 H 11 N 18	24.33	311.1409	3.7	3.7	63.4	8.5	even	ok
356.1974	1	C 20 H 26 N 3 O 3	100.00	356.1969	-1.5	-1.2	15.9	9.5	even	ok
	2	C 6 H 18 N 19	36.98	356.1987	3.7	0.9	39.8	7.5	even	ok
428.2083	1	C 25 H 26 N 5 O 2	100.00	428.2081	-0.5	-0.2	6.7	15.5	even	ok
	2	C 24 H 30 N O 6	36.20	428.2068	-3.6	-3.3	21.1	10.5	even	ok
	3	C 10 H 22 N 17 O 3	13.64	428.2086	0.7	0.3	76.3	8.5	even	ok
	4	C 13 H 30 N 7 O 9	6.06	428.2100	3.8	3.9	77.5	2.5	even	ok
	5	C 9 H 26 N 13 O 7	5.23	428.2073	-2.4	-2.4	90.3	3.5	even	ok
621.2725	1	C 36 H 37 N 4 O 6	56.73	621.2708	-2.8	-2.5	2.1	20.5	even	ok
	2	C 37 H 33 N 8 O 2	100.00	621.2721	-0.6	-0.4	14.0	25.5	even	ok
	3	C 25 H 37 N 10 O 9	22.88	621.2739	2.3	2.5	49.6	12.5	even	ok
	4	[illegible]	[illegible]	[illegible]	[illegible]	[illegible]	[illegible]	[illegible]	[illegible]	[illegible]

Figure S45. HRMS spectrum of **16**

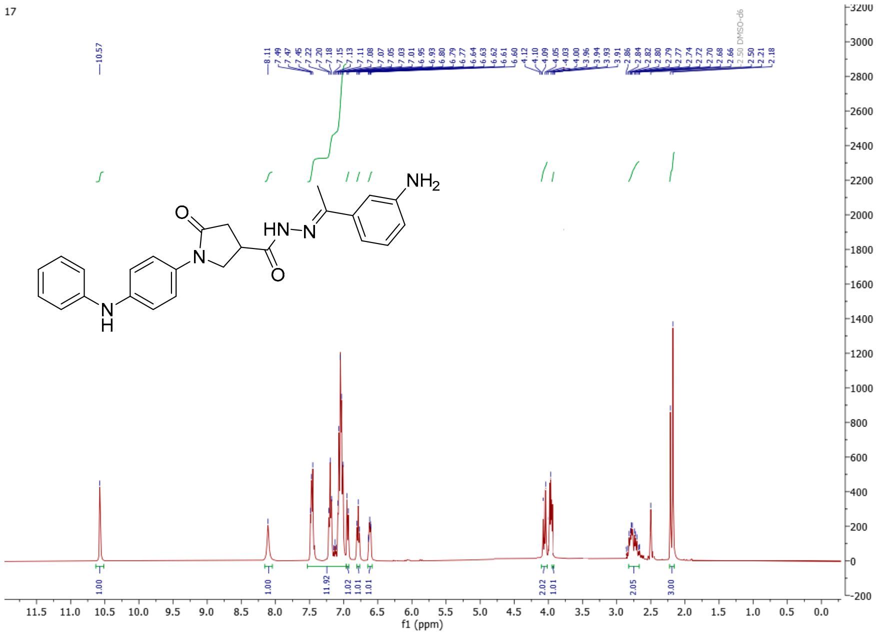

Figure S46. ^{1}H NMR (400 MHz, DMSO-d$_6$) spectrum of **17**

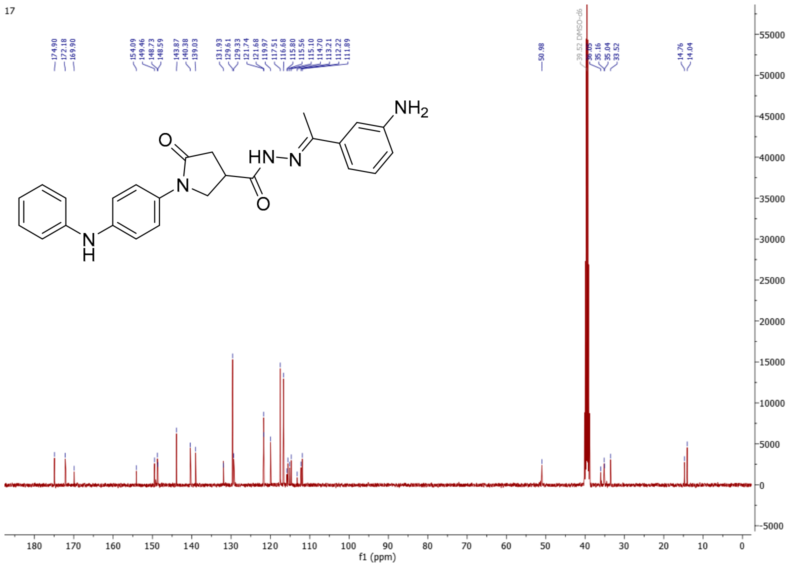

Figure S47. ^{13}C NMR (101 MHz, DMSO-d$_6$) spectrum of **17**

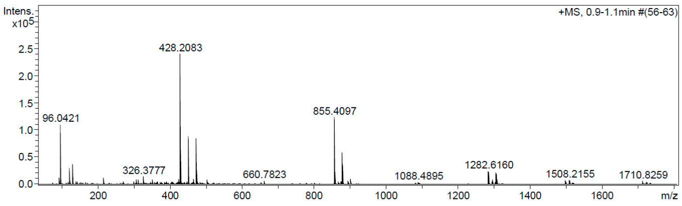

Meas. m/z	#	Formula	Score	m/z	err [ppm]	Mean err [ppm]	mSigma	rdb	e⁻ Conf	N-Rule
428.2083	1	C 24 H 30 N O 6	59.61	428.2068	-3.6	-3.3	1.7	10.5	even	ok
	2	C 25 H 26 N 5 O 2	100.00	428.2081	-0.5	-0.2	13.6	15.5	even	ok
	3	C 10 H 22 N 17 O 3	31.42	428.2086	0.7	0.4	56.4	8.5	even	ok
	4	C 13 H 30 N 7 O 9	14.08	428.2100	3.9	3.9	57.5	2.5	even	ok
	5	C 9 H 26 N 13 O 7	13.41	428.2073	-2.4	-2.4	70.2	3.5	even	ok
473.2661	1	C 27 H 33 N 6 O 2	100.00	473.2660	-0.4	-0.5	14.4	14.5	even	ok
	2	C 26 H 37 N 2 O 6	46.93	473.2646	-3.2	-2.9	15.8	9.5	even	ok
	3	C 15 H 37 N 8 O 9	8.26	473.2678	3.5	3.5	72.4	1.5	even	ok
	4	C 12 H 29 N 18 O 3	16.57	473.2665	0.7	0.2	74.4	7.5	even	ok
	5	C 11 H 33 N 14 O 7	7.37	473.2651	-2.1	-2.2	85.7	2.5	even	ok
855.4097	1	C 53 H 59 O 10	100.00	855.4103	0.6	1.0	4.7	24.5	even	ok
	2	C 50 H 51 N 10 O 4	83.42	855.4089	-1.0	-0.7	6.4	30.5	even	ok
	3	C 54 H 55 N 4 O 6	37.58	855.4116	2.2	2.5	13.7	29.5	even	ok
	4	C 51 H 47 N 14	80.37	855.4103	0.6	0.9	16.6	35.5	even	ok
	[illegible]	[illegible]	[illegible]	[illegible]	[illegible]	[illegible]	[illegible]	[illegible]	[illegible]	[illegible]

Figure S48. HRMS spectrum of **17**

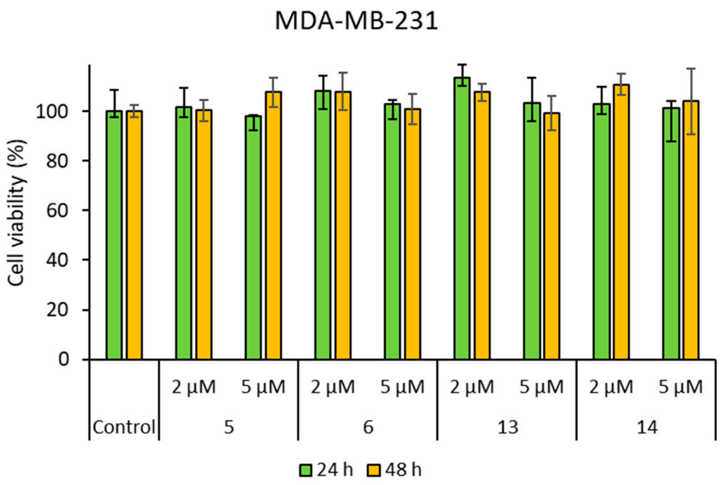

Figure S49. The effect of 2 and 5 µM compound concentration on MDA-MB-231 cell line viability after 24 and 48 h.

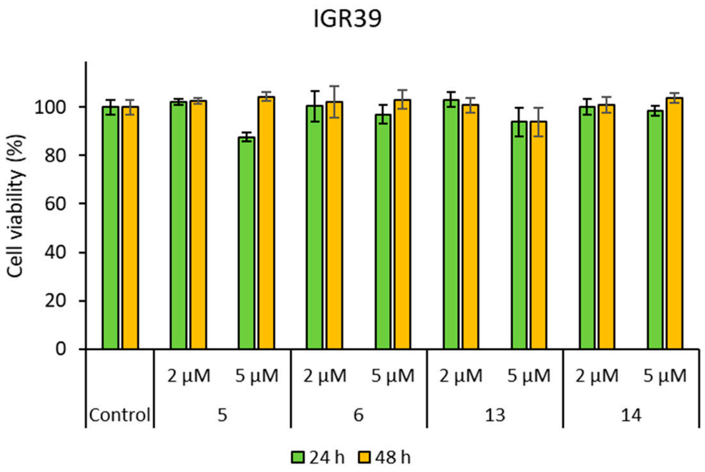

Figure S50. The effect of 2 and 5 µM compound concentration on IGR39 cell line viability after 24 and 48 h.

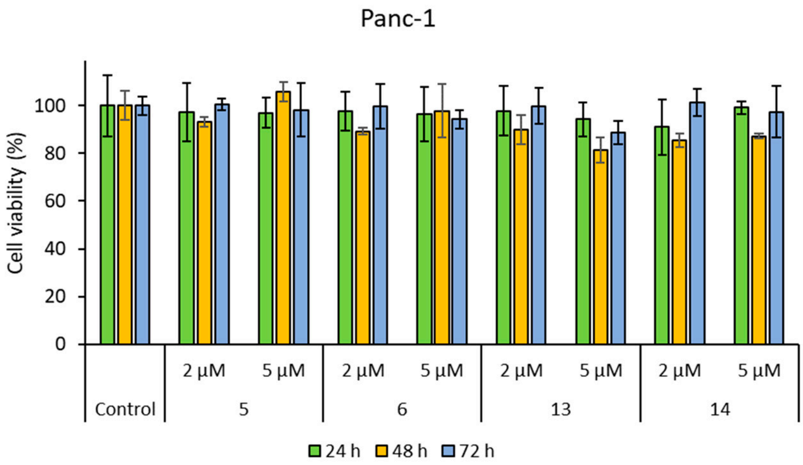

Figure S51. The effect of 2 and 5 µM compound concentration on Panc-1 cell line viability after 24, 48, and 72 h.

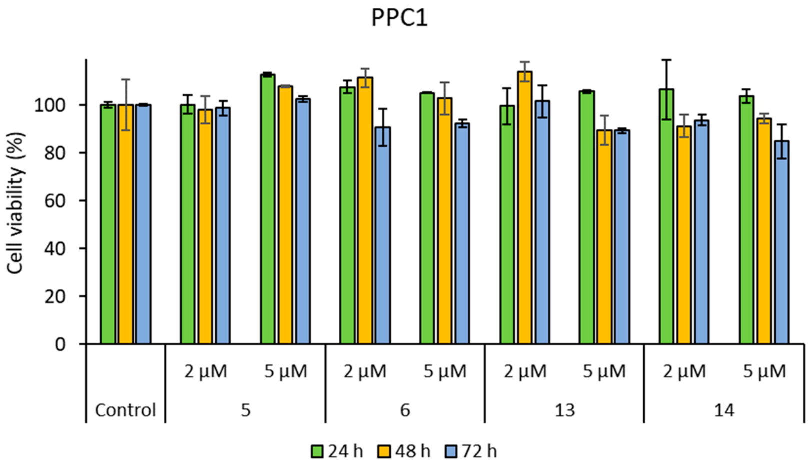

Figure S52. The effect of 2 and 5 µM compound concentration on PPC1 cell line viability after 24, 48, and 72 h.

Procedure S1. Evaluation of 2 and 5 µM compound concentration effect on cell line viability.

The effect of 10 µM compounds on cell viability was studied using 3-(4,5-dimethylthiazol-2-yl)-2,5-diphenyltetrazolium bromide (MTT; Sigma-Aldrich Co., St Louis, MO, USA) assay. IGR39, MDA-MB-231, Panc-1 and PPC1 cells were seeded in 96-well plates (Corning) in triplicates at a volume of 100 µL at 4×10^3 cells/well). After 24 h, the cells were treated with 2 and 5 µM of compounds **5, 6, 13,** and **14**. After 24 h, 48 h (all cell lines) and 72 h (Panc-1 and PPC1 cells), the MTT reagent has been added and cells were incubated for 4 h. Then the medium was aspirated, and the formed formazan crystals were dissolved in 100 µL DMSO (Sigma-Aldrich Co., St. Louis, MO, USA). The absorbance was measured at 570 and 630 nm using a multi-detection microplate reader. Compound effect on cell viability was calculated according to a formula:

$$Relative\ cell\ viability\ (\%) = \frac{A - A_0}{A_{NC} - A_0}$$

Where:

A – mean of absorbance of the tested compound,

A_0 – mean of absorbance of blank (no cells, positive control),

A_{NC} – mean of absorbance of negative control (only cells, no treatment).
